# OTUB1 non-canonically inhibits TAB2 ubiquitination to govern microglia-mediated neuroinflammation

**DOI:** 10.1038/s44321-026-00473-x

**Published:** 2026-07-02

**Authors:** Zijun Cao, Zhenhu Zhu, Ping Zeng, Fuqi Mei, Deqi Wang, Jun Xu, Yanqi Xu, Kangmin Chen, Chushan Wei, Jiangyun Shen, Keshuo Jin, Jiaqing Chen, Zhongding Li, Baohua Liu, Dirk Schlüter, Jingyong Huang, Xu Wang

**Affiliations:** 1 School of Pharmaceutical Sciences, Wenzhou Medical University, Wenzhou, China; 2 Oujiang Laboratory, Zhejiang Lab for Regenerative Medicine, Vision and Brain Health, Wenzhou, China; 3 The First School of Medicine and School of Information and Engineering, Wenzhou Medical University, Wenzhou, China; 4 Department of Rehabilitation, The First Affiliated Hospital of Wenzhou Medical University, Wenzhou, China; 5 Institute of Medical Microbiology and Hospital Epidemiology, Hannover Medical School, Hannover, Germany; 6 Department of Vascular Surgery, The First Affiliated Hospital of Wenzhou Medical University, Wenzhou, China

**Keywords:** Neuroscience

## Abstract

Microglia contribute to detrimental neuroinflammation under pathological conditions and thereby drive the pathogenesis and development of various diseases of the central nervous system (CNS). Here, the deubiquitinating enzyme OTUB1 is identified as a regulator of microglial activation and CNS inflammation. In mice, microglia-specific OTUB1 deletion significantly ameliorates ischemic brain injury by reducing the pro-inflammatory activation of microglia. OTUB1 enhances Toll-like receptor (TLR) signaling through stabilizing UBC13 and TAB2, leading to the increased induction of cytokines. Notably, OTUB1 reduces the proteasomal degradation of TAB2 by reducing its K48 ubiquitination in a catalytic activity-independent manner. Moreover, microglia-confined OTUB1 deficiency also alleviates lipopolysaccharide-induced sickness behavior and experimental autoimmune encephalomyelitis in mice due to decreased neuroinflammation. Pharmacological inhibition of OTUB1 significantly mitigated ischemic stroke injury in mice. These findings reveal an important role of OTUB1 in potentiating microglial activation and neuroinflammation, providing a proof-of-principle observation for targeting OTUB1 in the treatment of TLR-associated neuroinflammatory diseases.

## Synopsis

Pro-inflammatory activation of microglia under pathological conditions promotes the pathogenesis and progression of multiple neuroinflammatory diseases, such as ischemic stroke and multiple sclerosis. This study identifies OTUB1, a deubiquitinating enzyme, as a positive regulator of microglia-mediated neuroinflammation.

- Microglia-specific OTUB1 deficiency ameliorates neuroinflammatory diseases in mice.
- OTUB1 enhances the inflammatory response of microglia by stabilizing UBC13 and TAB2.
- Pharmacological inhibition of OTUB1 with a small-molecule inhibitor alleviates ischemic brain injury in mice.

## Abstract (web summary)

Pro-inflammatory activation of microglia under pathological conditions promotes the pathogenesis and progression of multiple neuroinflammatory diseases, such as ischemic stroke and multiple sclerosis. This study identifies OTUB1, a deubiquitinating enzyme, as a positive regulator of microglia-mediated neuroinflammation.

## Graphical abstract

**Figure:**
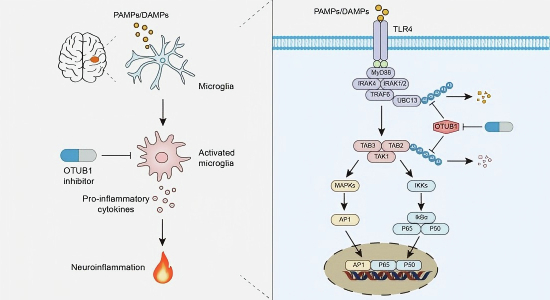

**Figure:**
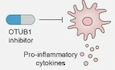

The paper explained **Problem**

Neuroinflammation is a common pathological feature of multiple brain diseases, such as ischemic stroke and multiple sclerosis. As important innate immune cells of the central nervous system (CNS), microglia mediate neuroinflammatory responses, thereby determining the progression of various brain diseases. The mechanisms regulating microglial activation remain incompletely understood.

**Results**

This study reveals that the pro-inflammatory activation of microglia requires the deubiquitinating enzyme OTUB1. Upon stimulation with danger signals, OTUB1 potentiates inflammatory signal transduction in microglia by increasing the expression of two pivotal signaling molecules, UBC13 and TAB2. Genetic or pharmacological manipulation of OTUB1 attenuates microglial activation and neuroinflammation in multiple CNS disease models.

**Impact**

These findings identify OTUB1 as a positive regulator of microglia-mediated neuroinflammation, providing a potential therapeutic target for neuroinflammatory diseases.

## Introduction

Microglia are key innate immune cells in the central nervous system (CNS) and are inextricably involved in the development, homeostasis, and diseases of the CNS (Fumagalli et al, 2025; Wolf et al, 2017). During development, microglia, which are generated before astrocytes and oligodendrocytes, actively participate in neurogenesis, synapse elimination, and the establishment of neural circuits (Frost and Schafer, 2016; Paolicelli et al, 2011). In addition, the immune surveillance and phagocytic scavenging function of microglia is critical for the maintenance of CNS homeostasis (Allen and Lyons, 2018; Prinz et al, 2019). Despite these beneficial functions, the pro-inflammatory activation of microglia under pathological conditions contributes to the pathogenesis and progression of various neuroinflammatory diseases, such as ischemic stroke, multiple sclerosis, CNS infections, and neurodegenerative diseases (Bartels et al, 2020; Charabati et al, 2023; Shi and Yong, 2025).

In the presence of CNS infection or tissue damage, microglia recognize danger signals, such as pathogen-associated molecular patterns (PAMPs) and damage-associated molecular patterns (DAMPs), by pattern recognition receptors (PRRs) (Prinz et al, 2019; Wang et al, 2020). Upon PRR activation, the intracellular nuclear factor-κB (NF-κB) and mitogen-activated protein kinase (MAPK) signaling pathways are activated to drive cytokine production, bestowing microglia a pro-inflammatory phenotype (Lou et al, 2025; Takeuchi and Akira, 2010). For example, DAMPs released from necroptotic neurons after cerebral stroke effectively activate Toll-like receptors (TLRs) on adjacent microglia to induce neuroinflammation, which further exacerbates the outcome of stroke (Huang et al, 2024). Similarly, microglia recognize PAMPs, such as lipopolysaccharide (LPS), by TLRs and subsequently produce pro-inflammatory cytokines (Sandiego et al, 2015; Sousa et al, 2018). Since dysregulated microglial activation propagates neuroinflammatory responses and causes neuropathology, PRR-mediated signal transduction is tightly controlled by exquisite mechanisms including modification of signal molecules by ubiquitination.

As a reversible post-translational modification, ubiquitination is catalyzed in a hierarchical manner by E1, E2, and E3 ubiquitinating enzymes and inhibited by deubiquitinating enzymes (DUBs) (Huang et al, 2024; Liu et al, 2022; Ruan et al, 2022). Accumulating studies have revealed the pivotal role of ubiquitin-modifying enzymes in the regulation of microglial activation. The E3 ubiquitin ligase COP1 suppresses tau-mediated neurodegeneration in mice by inhibiting microglia-mediated neuroinflammation (Ndoja et al, 2020). On the contrary, the E3 ligase Peli1 enhances TLR signaling by inducing the ubiquitination-dependent degradation of TNF receptor-associated factor 3 (TRAF3), thereby promoting microglia-mediated neuroinflammation in experimental autoimmune encephalomyelitis (EAE) (Xiao et al, 2013). Moreover, the DUBs DUBA and USP25 regulate CNS inflammation by enhancing and suppressing TLR-triggered signaling, respectively (Li et al, 2023; Zhu et al, 2025). Although DUBs may identically augment or inhibit the same signaling pathway under pathological conditions, their function is largely non-redundant and the individual DUBs cannot compensate for each other.

OTUB1 is a DUB that is intricately related to inflammation. Previously, we and others have found that the whole-body deletion of OTUB1 leads to embryonic lethality in mice (Pasupala et al, 2018; Ruiz-Serrano et al, 2021; Wang et al, 2019). Using cell-specific or inducible OTUB1 knockout mice, studies have elucidated key functions of OTUB1 in regulating immune and inflammatory responses. Deletion of OTUB1 strengthens anticancer immunity by increasing the sensitivity of CD8^+^ T cells and natural killer (NK) cells to IL-15 (Zhou et al, 2019). In addition, OTUB1 prevents the aberrant NF-κB activation in B cells via stabilizing p100, and B cell-specific OTUB1 deletion leads to lupus-like autoimmunity in mice (Li et al, 2019). We have shown that OTUB1 deficiency in dendritic cells impairs TLR-induced IL-12 production, rendering mice incapable of initiating an effective immune response against Toxoplasma gondii infection (Mulas et al, 2021). However, the role of OTUB1 in microglia remains elusive.

In this study, we discovered a new function of OTUB1 in modulating microglial activation and neuroinflammation. Conditional ablation of OTUB1 in microglia significantly ameliorated ischemic stroke injury, LPS-induced sickness behavior, and EAE by mitigating CNS inflammation in mice. OTUB1 enhanced TLR signaling by increasing the protein abundance of UBC13 and TAB2, with the latter being a newly identified protein target of OTUB1. Mechanistically, OTUB1 reduced the K48 ubiquitination of TAB2 in a non-canonical manner, leading to the reduced degradation of TAB2 in the proteasome. Furthermore, pharmacological inhibition of OTUB1 significantly attenuated ischemic stroke injury in mice, accompanied by reduced microglial activation. Therefore, our study identifies OTUB1 as a regulator of microglia in CNS inflammation.

## Results

### Genetic inhibition of microglial OTUB1 reduces ischemic brain injury

**Figure 1:**
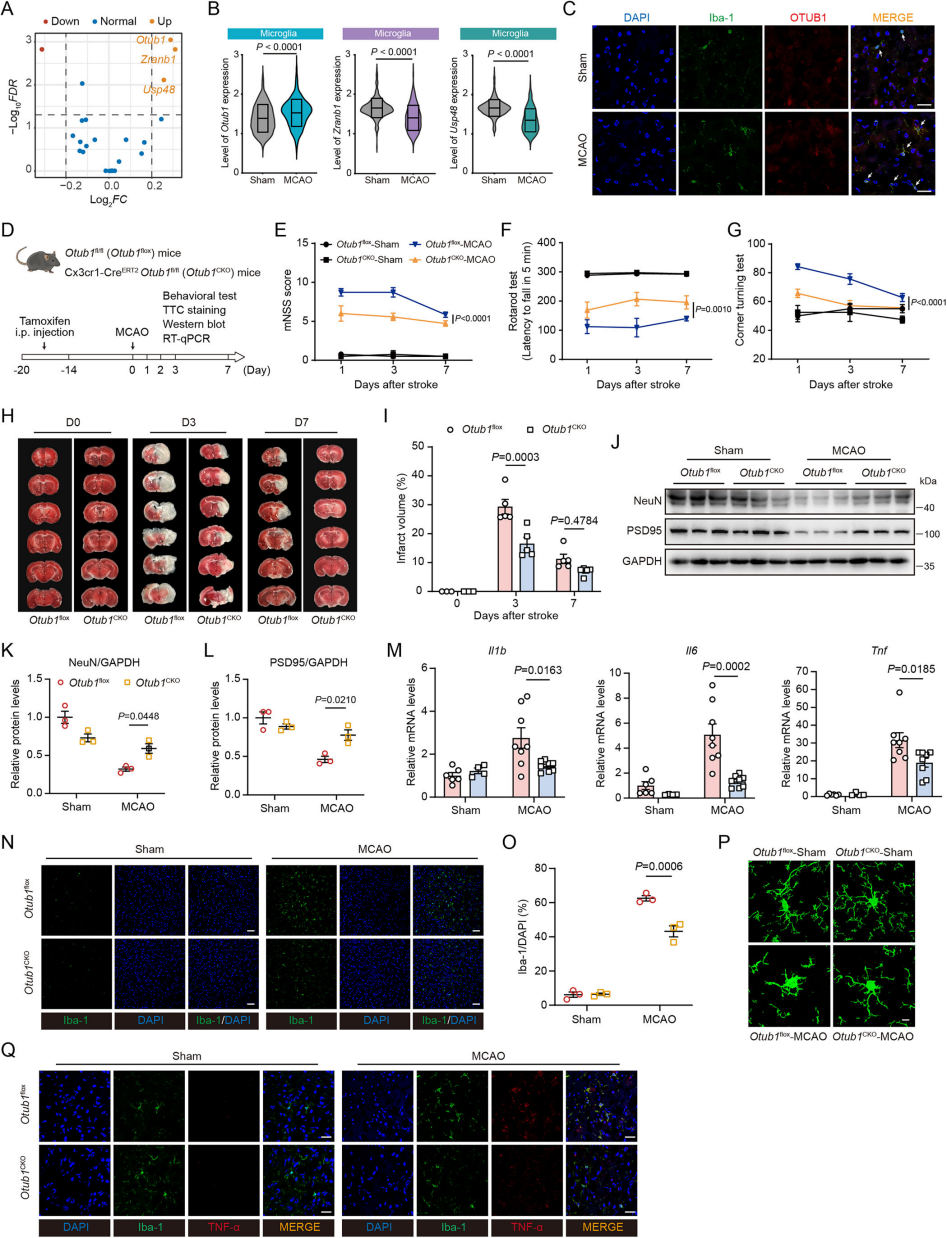
Microglia-specific OTUB1 deficiency ameliorates MCAO-induced cerebral damage in mice. (**A**) Volcano plot showing the differential expression of DUBs in the peripheral whole blood of the control group (*n* = 240, biological replicates) and patients with ischemic stroke (*n* = 39, biological replicates) based on GSE16561. (**B**) Violin plot showing the expression levels of *Otub1* (*n* = 4876–6092, biological replicates), *Zranb1* (*n* = 502–533, biological replicates), and *Usp48* (*n* = 402–539, biological replicates) in microglia of control mice and mice with ischemic stroke based on GSE174574. (**C**) C57BL/6 mice were treated with sham operation or transient MCAO for 70 min. On day 2 after MCAO, brain sections were immunostained for Iba-1 and OTUB1. Scale bar = 50 µm. (**D**) Schematic diagram showing the timeline for MCAO experiments. (**E**–**G**) On days 1, 3, and 7 after MCAO, mice were evaluated by the mNSS (**E**), rotarod test (**F**), and corner turning test (**G**) (*n* = 3–7, biological replicates). (**H**, **I**) On days 3 and 7 after MCAO, cerebral infarction was assessed by TTC staining. Representative images (**H**) and infarct volume (**I**) are shown (*n* = 3–5, biological replicates). (**J**–**L**) The expression of NeuN and PSD95 proteins in the brain of indicated mice on day 2 after MCAO was detected by immunoblotting. Representative immunoblots (**J**) as well as quantification for NeuN (**K**) and PSD95 (**L**) are shown (*n* = 3, biological replicates). (**M**) Transcriptional levels of *Il1b*, *Il6*, and *Tnf* in the brain of indicated mice on day 2 after MCAO were determined by qPCR (*n *= 4–8, biological replicates). (**N**, **O**) Brain sections of indicated mice on day 2 after MCAO were immunostained for Iba-1. Representative images (**N**) and quantification (**O**) are shown (*n* = 3, biological replicates). Scale bar =  50 µm. (**P**) Representative Z-stack images of Iba-1 immunostaining in the ischemic penumbra of indicated mice on day 2 after MCAO. Scale bar = 5 µm. (**Q**) Brain sections of indicated mice on day 2 after MCAO were immunostained for Iba-1 and TNF-α. Scale bar = 50 µm. Data in (**B**) show mean ± SD and data in (**E**–**G**, **I**, **K**–**M**, **O**) represent mean ± SEM. Statistics were performed using the Mann–Whitney *U* test (**B**) and two-way ANOVA test (**E**–**G**, **I**, **K**–**M**, **O**). Source data are available online for this figure.

To identify DUBs that participate in cerebral ischemic injury, we examined the expression levels of DUBs in the peripheral whole blood and found that the expression of *Otub1*, *Zranb1*, and *Usp48* was significantly elevated in patients suffering from ischemic stroke compared with the control group (Fig. 1A). Subsequently, we analyzed the expression of *Otub1*, *Zranb1*, and *Usp48* in different cell populations in the mouse brain and found that the expression of *Otub1*, but not *Zranb1* and *Usp48*, was significantly upregulated in microglia of mice treated with middle cerebral artery occlusion (MCAO) (Fig. 1B; Appendix Fig. S1A). Moreover, expression of *Otub1* was not significantly changed in astrocytes of MCAO mice (Appendix Fig. S1B). Furthermore, we verified that OTUB1 was highly upregulated in Iba-1^+^ microglia in the ischemic penumbra of MCAO-treated mice (Fig. 1C). To elucidate the role of microglia-derived OTUB1 in ischemic brain injury, we generated Cx3cr1-Cre^ERT2^ *Otub1*^fl/fl^ (*Otub1*^CKO^) mice (Appendix Fig. S2A). After tamoxifen treatment, OTUB1 was selectively and efficiently deleted in microglia of *Otub1*^CKO^ mice (Fig. 1D; Appendix Fig. S2B–H). Interestingly, deletion of microglial OTUB1 significantly ameliorated MCAO-induced neurological deficits, as revealed by modified neurological severity score (mNSS), rotarod test, and corner turning test (Fig. 1E–G). Consistently, on days 3 and 7 after MCAO, the brain infarct volume in *Otub1*^CKO^ mice was significantly smaller than that in *Otub1*^flox^ mice (Fig. 1H,I). Furthermore, immunoblot analysis found that MCAO-induced downregulation of NeuN and PSD95 was attenuated in *Otub1*^CKO^ mice (Fig. 1J–L), demonstrating that the specific ablation of OTUB1 in microglia ameliorates neuronal damage after ischemic brain insult. In addition, significantly more NeuN^+^ neurons were detected in the ischemic penumbra of *Otub1*^CKO^ mice compared with *Otub1*^flox^ mice (Appendix Fig. S3A,B). Microglia contribute to neuroinflammation after cerebral ischemic insult by producing proinflammatory cytokines (Li et al, 2023; Zhu et al, 2025). Compared with *Otub1*^flox^ mice, *Otub1*^CKO^ mice had significantly lower levels of *Il1b*, *Il6*, and *Tnf* mRNA in the infarcted brain (Fig. 1M), implicating that OTUB1 promotes cerebral ischemic injury by enhancing microglia-mediated neuroinflammation. Indeed, both the quantity and activation of microglia were decreased in the ischemic penumbra in the absence of microglial OTUB1 (Fig. 1N–P). Furthermore, immunofluorescence staining showed that OTUB1 deletion reduced TNF-α production in microglia in the ischemic penumbra (Fig. 1Q). Collectively, these results demonstrate that ischemic brain injury is ameliorated in *Otub1*^CKO^ mice, accompanied by reduced neuroinflammation and microglial activation, indicating that microglia-derived OTUB1 promotes ischemic brain injury by potentiating microglia-mediated neuroinflammatory responses.

### OTUB1 enhances the pro-inflammatory activation of microglia

**Figure 2:**
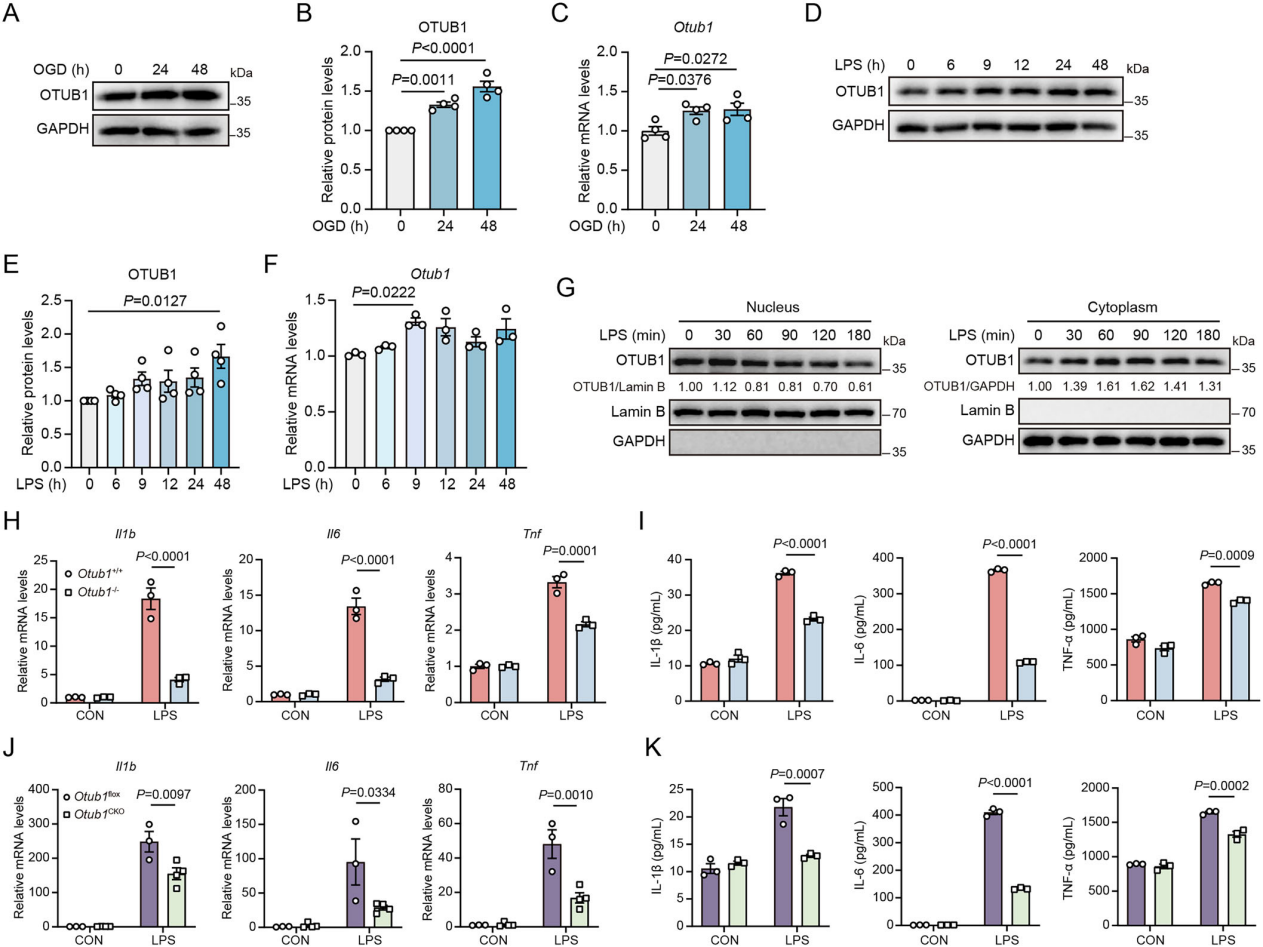
OTUB1 deficiency reduces the production of pro-inflammatory cytokines in microglia. (**A**, **B**) BV2 cells were subjected to OGD for 6 h, followed by reoxygenation for 24 and 48 h. Cell lysates were analyzed by immunoblotting for OTUB1. Representative immunoblots (**A**) and quantification (**B**) are shown (*n* = 4, biological replicates). (**C**) BV2 cells were treated with OGD for 6 h, followed by reoxygenation for 24 and 48 h. Transcriptional levels of *Otub1* were determined by qRT-PCR (*n* = 4, biological replicates). (**D**, **E**) Immunoblot analysis of OTUB1 in BV2 cells treated with LPS (500 ng/mL) for the indicated time periods. Representative immunoblots (**D**) and quantification (**E**) are shown (*n* = 4, biological replicates). (**F**) Transcriptional levels of *Otub1* in BV2 cells treated with LPS (500 ng/mL) for the indicated time periods were determined by qRT-PCR (*n* = 3, biological replicates). (**G**) BV2 cells were treated with LPS (500 ng/mL) for the indicated time periods. The cytoplasmic and nucleic fractions were separated and then analyzed by immunoblotting for OTUB1. (**H**) Transcriptional levels of *Il1b*, *Il6*, and *Tnf* in BV2 cells stimulated with LPS (500 ng/mL) for 3 h were analyzed by qRT-PCR (*n* = 3, biological replicates). (**I**) BV2 cells were stimulated with LPS (500 ng/mL) for 12 h. Protein levels of IL-1β, IL-6, and TNF-α in the supernatant were analyzed by ELISA (*n* = 3, biological replicates). (**J**) Transcriptional levels of *Il1b*, *Il6*, and *Tnf* in primary microglia stimulated with LPS (500 ng/mL) for 3 h were analyzed by qRT-PCR (*n* = 3–4, biological replicates). (**K**) Primary microglia were stimulated with LPS (500 ng/mL) for 12 h. Protein levels of IL-1β, IL-6, and TNF-α in the supernatant were analyzed by ELISA (*n* = 3, biological replicates). Data in (**B**, **C**, **E**, **F**, **H**–**K**) represent mean ± SEM. Statistics were performed with the one-way (**B**, **C**, **E**, **F**) or two-way (**H**–**K**) ANOVA test. Source data are available online for this figure.

In line with the in vivo observation that OTUB1 expression was increased in microglia after ischemic insult (Fig. 1B), we found that OTUB1 was also significantly upregulated in immortalized BV2 microglial cells after treatment with oxygen-glucose deprivation (OGD) at both mRNA and protein levels (Fig. 2A–C). Shortly after MCAO, microglia are activated by DAMPs released from necroptotic cells, leading to the production of cytokines (Naito et al, 2020). To simulate the inflammatory activation of microglia in vitro, BV2 cells were treated with LPS. Similar to OGD treatment, LPS stimulation significantly upregulated OTUB1 expression in BV2 cells (Fig. 2D–F). OTUB1 is a DUB that resides in both the cytosol and nucleus (Herhaus et al, 2015; Wang et al, 2019). Intriguingly, short-term simulation with LPS induced the nucleus-to-cytoplasm translocation of OTUB1 in BV2 cells (Fig. 2G), indicating that OTUB1 may affect LPS-induced signal transduction in the cytoplasm of microglia. To study the role of OTUB1 in the inflammatory activation of microglia, we generated *Otub1*^−/−^ BV2 cells and found that *Otub1*^−/−^ BV2 cells produced significantly less IL-1β, IL-6, and TNF-α at both transcriptional and protein levels compared with control BV2 cells upon LPS stimulation (Fig. 2H,I). Similarly, OTUB1 deletion also significantly impaired the ability of primary microglia to produce pro-inflammatory cytokines (Fig. 2J,K), indicating that OTUB1 contributes to the full-fledged activation of microglia upon pro-inflammatory stimulation.

### OTUB1 enhances LPS-induced microglial activation by increasing TAB2 expression

**Figure 3:**
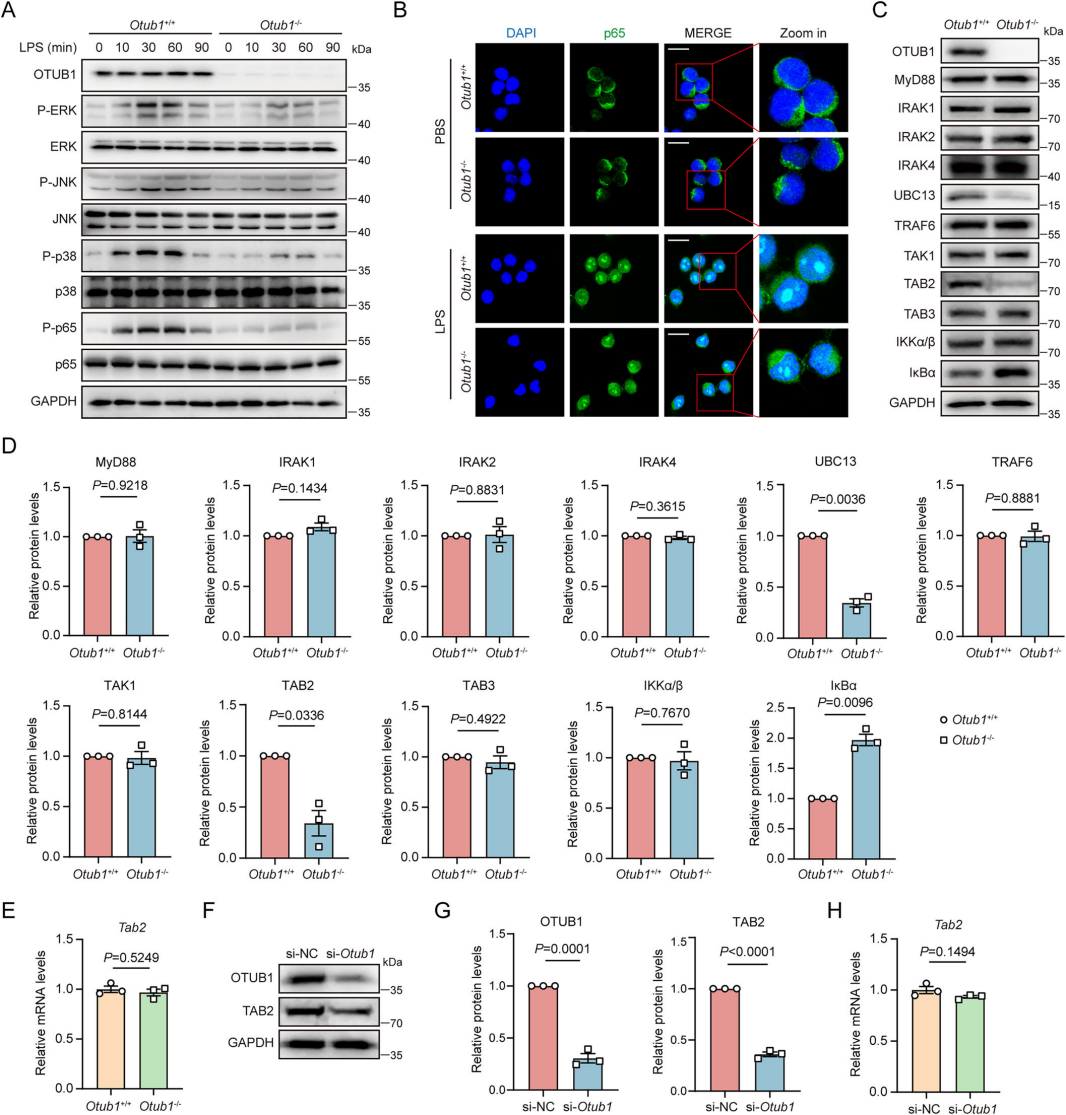
OTUB1 deficiency impairs LPS-induced signal transduction by reducing the protein abundance of TAB2. (**A**) Immunoblot analysis of the indicated signaling molecules in *Otub1*^+/+^ and *Otub1*^−/−^ BV2 cells stimulated with LPS (500 ng/mL) for 0, 10, 30, 60, and 90 min. (**B**) *Otub1*^+/+^ and *Otub1*^−/−^ BV2 cells stimulated with LPS (500 ng/mL) for 0 and 30 min were immunostained for p65. Scale bar = 20 µm. (**C**, **D**) Whole-cell lysates of *Otub1*^+/+^ and *Otub1*^−/−^ BV2 cells were analyzed by immunoblotting for the indicated signaling molecules. Representative immunoblots (**C**) and quantification (**D**) are shown (*n* = 3, biological replicates). (**E**) Transcriptional levels of *Tab2* in *Otub1*^+/+^ and *Otub1*^−/−^ BV2 cells were determined by qRT-PCR (*n* = 3, biological replicates). (**F**, **G**) Immunoblot analysis of OTUB1 and TAB2 in BV2 cells transfected with si-NC (negative control) or si-*Otub1* for 48 h. Representative immunoblots (**F**) and quantification (**G**) are shown (*n* = 3, biological replicates). (**H**) Transcriptional levels of *Tab2* in BV2 cells transfected with si-NC or si-*Otub1* for 48 h were determined by qRT-PCR (*n* = 3, biological replicates). Data in (**D**, **E**, **G**, **H**) are expressed as mean ± SEM. Statistics were analyzed with the two-tailed Student’s *t* test. Source data are available online for this figure.

To parse out the mechanism of OTUB1 in regulating microglial activation, we analyzed key signaling molecules in LPS-stimulated *Otub1*^+/+^ and *Otub1*^−/−^ BV2 cells and found that OTUB1 deletion strongly diminished the phosphorylation of p65, p38, JNK, and ERK (Fig. 3A), indicating that OTUB1 enhances NF-κB and MAPK signaling in activated microglia. Moreover, immunofluorescence staining revealed that OTUB1 deficiency impaired the nuclear translocation of p65 in BV2 cells after LPS stimulation (Fig. 3B). Given that OTUB1 preferentially cleaves K48-specific polyubiquitin chains and that K48 ubiquitination induces the proteasomal degradation of protein substrates (Mevissen et al, 2013), we analyzed the impact of OTUB1 deletion on the protein abundance of critical signaling molecules in MAPK and NF-κB signaling pathways (Fig. 3C; Appendix Fig. S4). *Otub1*^−/−^ BV2 cells had significantly lower levels of UBC13 and TAB2 compared with *Otub1*^+/+^ BV2 cells (Fig. 3C,D). In line with our results, OTUB1 was previously identified to stabilize UBC13 in dendritic cells by modulating K48 ubiquitination (Mulas et al, 2021). Furthermore, we observed that OTUB1 deletion had no discernible influence on the mRNA levels of *Tab2* (Fig. 3E), suggesting that OTUB1 may also regulate TAB2 protein abundance at post-translational levels. Moreover, knockdown of *Otub1* with siRNA significantly decreased TAB2 protein expression in BV2 cells without affecting *Tab2* mRNA expression (Fig. 3F–H), verifying that TAB2 and OTUB1 protein levels are positively correlated. Therefore, these findings indicate that OTUB1 potentiates LPS-induced NF-κB and MAPK activation by elevating the protein levels of UBC13 and TAB2.

### OTUB1 inhibits the degradation of TAB2 through the proteasome

**Figure 4:**
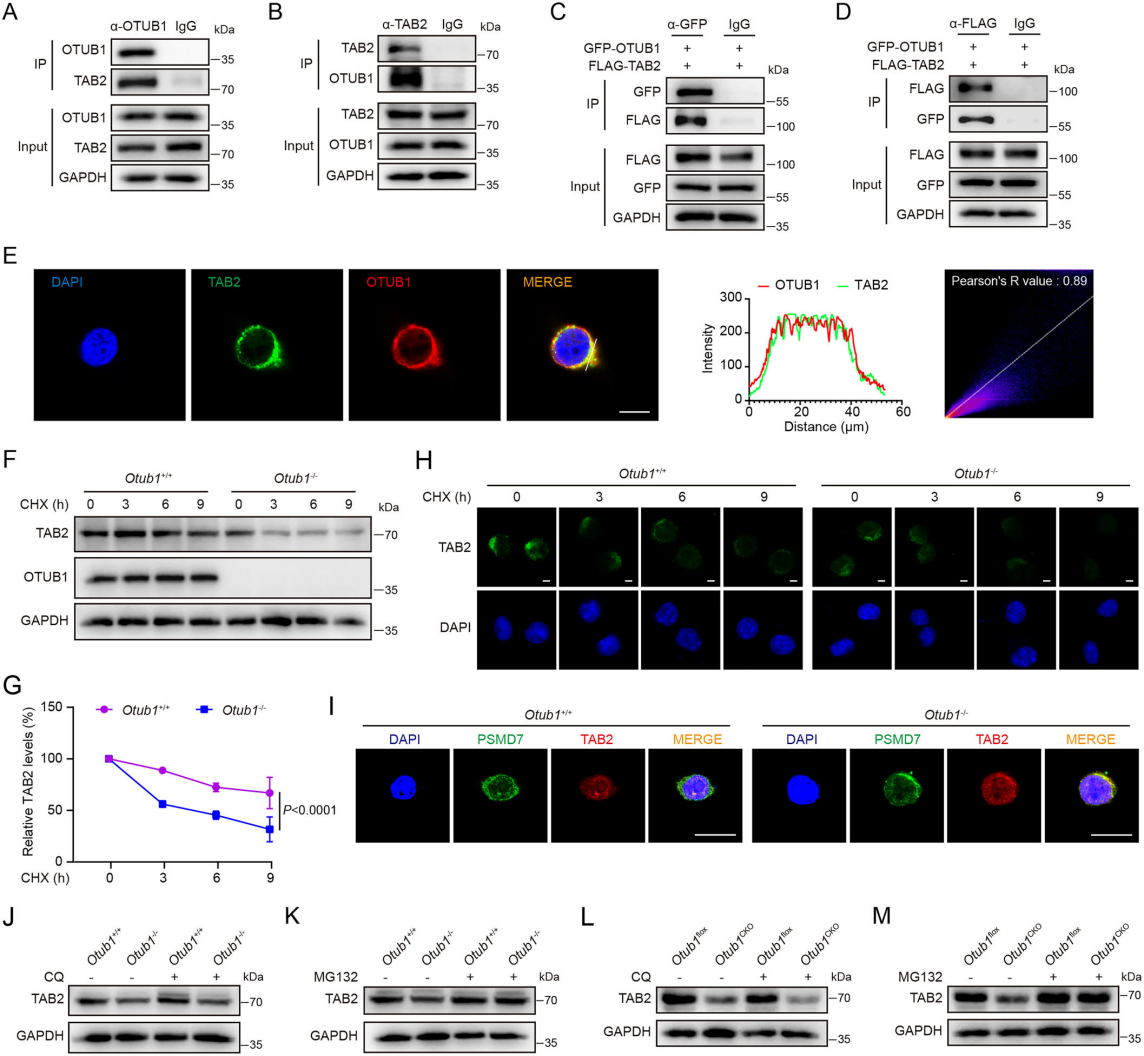
OTUB1 interacts with TAB2 and reduces the proteasomal degradation of TAB2. (**A**, **B**) BV2 cell lysates were immunoprecipitated with anti-OTUB1 (**A**) or anti-TAB2 (**B**) antibodies, followed by immunoblot analysis. IgG served as negative control. (**C**, **D**) BV2 cells were co-transfected with GFP-OTUB1 and FLAG-TAB2 plasmids for 24 h before lysis. Cell lysates were immunoprecipitated with anti-GFP (**C**) and anti-FLAG (**D**) antibodies, followed by immunoblot analysis. (**E**) BV2 cells were immunostained for OTUB1 and TAB2 (left panel). The middle and right panels show the fluorescence intensity and Pearson’s correlation coefficient, respectively. Scale bar = 10 µm. (**F**, **G**) Immunoblot analysis of TAB2 in *Otub1*^+/+^ and *Otub1*^−/−^ BV2 cells treated with CHX (10 µM) for the indicated time periods. Representative immunoblots (**F**) and quantification (**G**) are shown (*n* = 3, biological replicates). (**H**) *Otub1*^+/+^ and *Otub1*^−/−^ BV2 cells treated with CHX (10 µM) for the indicated time periods were immunostained for TAB2. Scale bar = 5 µm. (**I**) *Otub1*^+/+^ and *Otub1*^−/−^ BV2 cells were immunostained for TAB2 and PSMD7. Scale bar =  20 µm. (**J**, **K**) Immunoblotting for TAB2 in *Otub1*^+/+^ and *Otub1*^−/−^ BV2 cells treated with 10 µM of CQ (**J**) or 5 µM of MG132 (**K**) for 0 and 6 h. (**L**, **M**) Immunoblotting for TAB2 in primary microglia from *Otub1*^flox^ and *Otub1*^CKO^ mice treated with 10 µM of CQ (**L**) or 5 µM of MG132 (**M**) for 0 and 6 h. Data in (**G**) are expressed as mean ± SEM. Statistics were analyzed with the two-way ANOVA test. Source data are available online for this figure.

Given that OTUB1 has already been shown to inhibit the proteasomal degradation of UBC13 by regulating K48 ubiquitination (Mulas et al, 2021), we proceeded to analyze the molecular mechanism applied by OTUB1 to regulate TAB2. Co-immunoprecipitation and immunoblotting showed that OTUB1 interacted with TAB2 at both endogenous and exogenous levels (Fig. 4A–D). Consistently, immunofluorescence staining found that OTUB1 co-localized with TAB2 in BV2 cells (Fig. 4E). Since OTUB1 had no impact on *Tab2* mRNA levels (Fig. 3E,H), it is possible that OTUB1 enhanced TAB2 protein expression by reducing TAB2 degradation. Indeed, OTUB1 deficiency accelerated the degradation of TAB2 in BV2 cells, as revealed by the cycloheximide (CHX) chase assay (Fig. 4F–H). Proteins are degraded mainly through the autophagy-lysosomal pathway or the ubiquitin-proteasome system (Liu et al, 2022). OTUB1 deficiency intensified the co-localization of TAB2 with the proteasome marker PSMD7 but could not induce the co-localization of TAB2 with the lysosome marker LAMP1 (Fig. 4I; Appendix Fig. S5), indicating that OTUB1 hinders the degradation of TAB2 in the proteasome. In good agreement, treating with the proteasome inhibitor MG132, but not the lysosome inhibitor chloroquine, elevated TAB2 protein abundance in *Otub1*^+/+^ and *Otub1*^−/−^ BV2 cells to comparable levels (Fig. 4J,K). Consistently, OTUB1 inhibited the proteasomal degradation of TAB2 in primary microglia (Fig. 4L,M). Together, these data demonstrate that OTUB1 interacts with TAB2 and inhibits its degradation through the proteasome.

### OTUB1 impedes the K48 ubiquitination of TAB2 in a non-canonical manner

**Figure 5:**
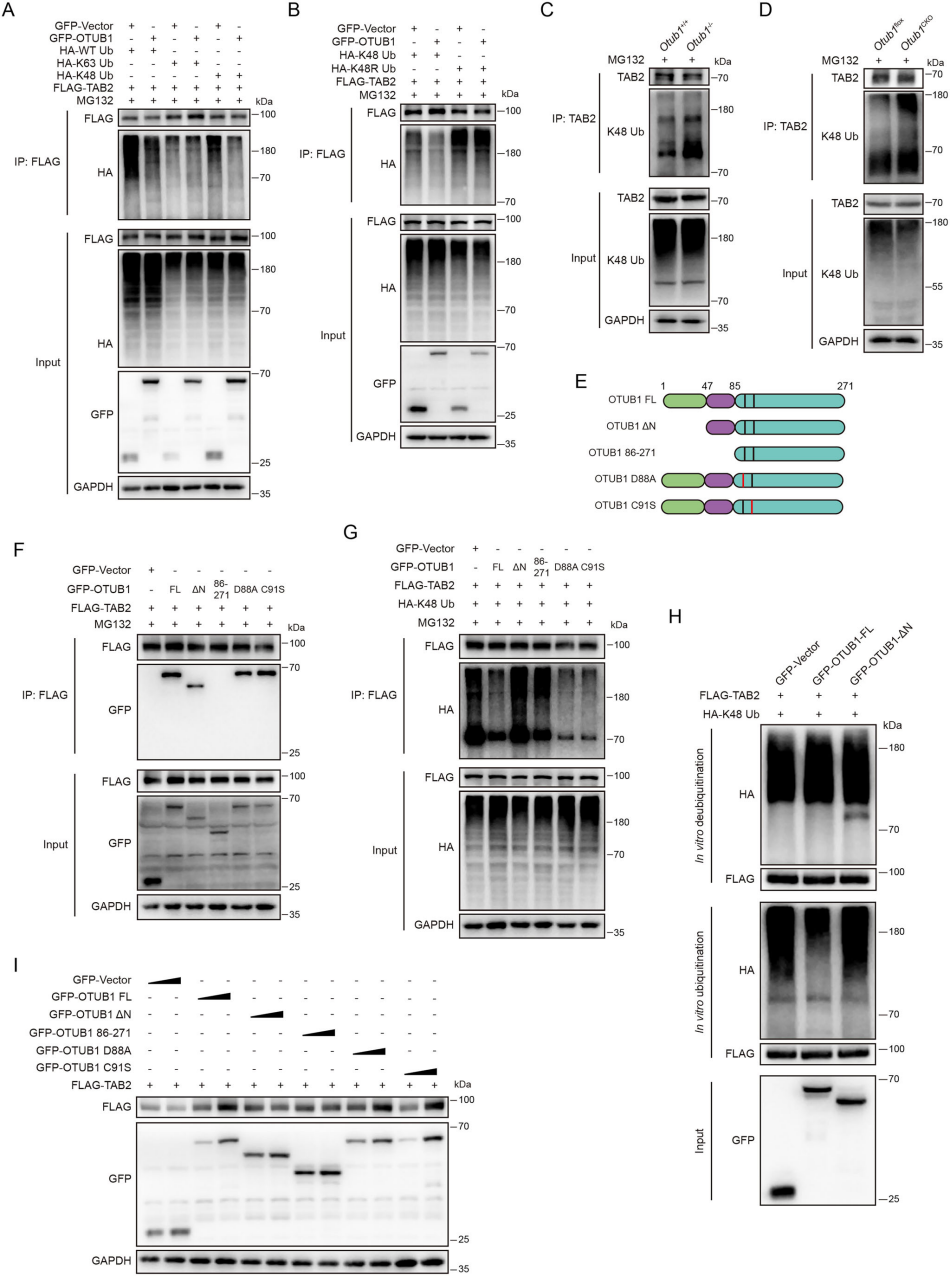
OTUB1 inhibits the K48 ubiquitination of TAB2 through the N-terminus. (**A**, **B**) NIH/3T3 cells were transfected with indicated plasmids for 24 h and then treated with MG132 (20 µM) for 6 h. Cell lysates were immunoprecipitated with anti-FLAG antibody, followed by immunoblot analysis. (**C**) Whole-cell lysates of *Otub1*^+/+^ and *Otub1*^−/−^ BV2 cells were immunoprecipitated with anti-TAB2 antibody, followed by immunoblot analysis. (**D**) Whole-cell lysates of primary microglia from *Otub1*^flox^ and *Otub1*^CKO^ mice were immunoprecipitated with anti-TAB2 antibody, followed by immunoblot analysis. (**E**) Schematic of OTUB1 and its mutants. (**F**, **G**) NIH/3T3 cells were transfected with indicated plasmids for 24 h and then treated with MG132 (20 µM) for 6 h. Cell lysates were immunoprecipitated with anti-FLAG antibody, followed by immunoblot analysis. (**H**) The effect of OTUB1 on the ubiquitination and deubiquitination of TAB2 was analyzed by the in vitro ubiquitination and deubiquitination assays, respectively. (**I**) Immunoblot analysis of NIH/3T3 cells transfected with indicated plasmids for 24 h. Source data are available online for this figure.

It has been reported that K48- and K63-linked polyubiquitin chains can be attached to TAB2 (Kanayama et al, 2004; Xia et al, 2009; Zhang et al, 2023). We identified that OTUB1 specifically reduced K48, rather than K63, polyubiquitination of TAB2 (Fig. 5A), which is consistent with previous studies that the proteasomal degradation of TAB2 is triggered by K48 ubiquitination (Hou et al, 2021; Tian et al, 2007; Zhang et al, 2023). Moreover, OTUB1 failed to reduce polyubiquitination linked by the K48R (lysine 48 mutated to arginine) mutant of ubiquitin (Fig. 5B), consolidating the specificity of OTUB1 toward K48 ubiquitination on TAB2. Consistently, at endogenous levels, OTUB1 deletion markedly increased K48 ubiquitination of TAB2 in BV2 cells and primary microglia (Fig. 5C,D). OTUB1 comprises a ubiquitin-binding domain, a linker domain, and an OTU domain (Herhaus et al, 2013; Messick et al, 2008; Zhao et al, 2023). In the OTU domain, the catalytic residue C91 mediates the deubiquitinating activity of OTUB1, and the D88 site exerts a non-canonical ubiquitination-inhibiting function by inhibiting E2 activity (Fig. 5E) (Juang et al, 2012; Sun et al, 2012; Wu et al, 2021). Co-immunoprecipitation experiment revealed that OTUB1 interacted with TAB2 through the linker domain (Fig. 5F). Since the 86-271 mutant could not interact with TAB2, it was also unable to modulate TAB2 ubiquitination (Fig. 5G). Interestingly, both the D88A and C91S mutants of OTUB1 still possessed the ability to reduce K48 ubiquitination of TAB2, whereas the ∆N mutant failed to reduce TAB2 ubiquitination (Fig. 5G). Notably, the N-terminus of OTUB1 is also required for the non-canonical function of OTUB1 to impede ubiquitination of substrates (Wang et al, 2019; Wiener et al, 2012). Indeed, OTUB1 was found to hamper the ubiquitination of TAB2 but failed to deubiquitinate TAB2 in the in vitro ubiquitination and deubiquitination assays (Fig. 5H). Therefore, instead of cleaving ubiquitin chains on TAB2 through its deubiquitinating activity, OTUB1 reduced K48 ubiquitination of TAB2 by blocking ubiquitination. Furthermore, unlike the D88A and C91S mutants, which stabilized TAB2 as efficiently as the wild-type OTUB1, the ∆N mutant lost the ability to stabilize TAB2 (Fig. 5I). Taken together, these data exhibit that OTUB1 non-canonically inhibits the K48 ubiquitination of TAB2 through the N-terminal UBA domain, thereby enhancing TAB2 stability.

### OTUB1 potentiates ischemic brain injury by regulating TAB2

**Figure 6:**
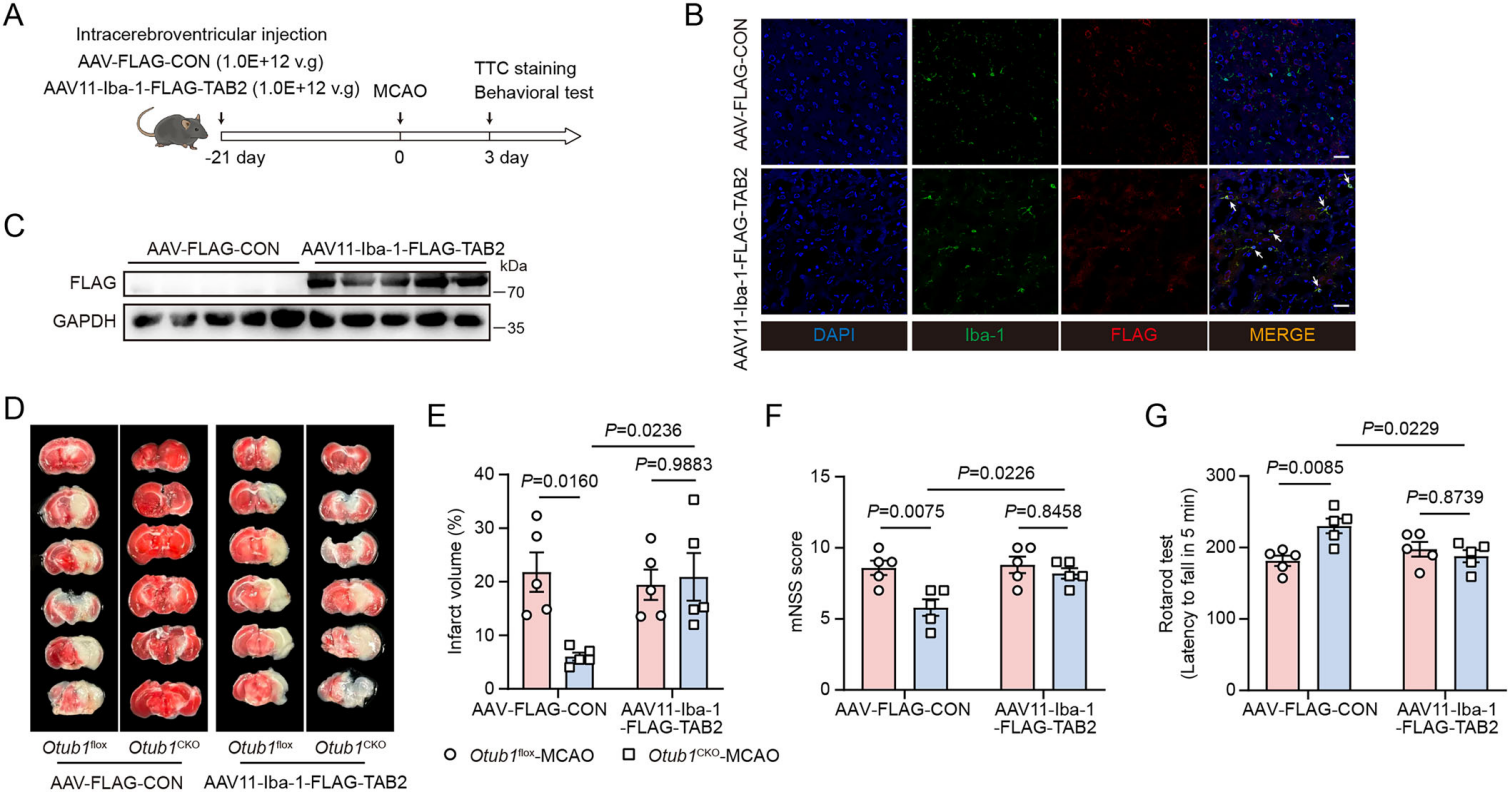
Microglial OTUB1 enhances ischemic brain injury by regulating TAB2. (**A**) Schematic diagram showing the timeline for AAV injection and MCAO experiments. (**B**) Brain sections of AAV-treated *Otub1*^flox^ mice were immunostained with Iba-1 and FLAG. (**C**) FLAG-TAB2 expression in brain tissues of AAV-treated *Otub1*^flox^ mice was analyzed by western blot. (**D**,** E**) Cerebral infarction of indicated mice was assessed by TTC staining. Representative images (**D**) and infarct volume (**E**) are shown (*n* = 5, biological replicates). (**F**, **G**) Mice were evaluated by the mNSS (**F**) and rotarod test (**G**) (*n* = 5, biological replicates). Data in (**E**–**G**) represent mean ± SEM. Statistics were performed using the two-way ANOVA test. Source data are available online for this figure.

Our in vitro experiments demonstrated that OTUB1 enhanced TLR-mediated inflammatory responses by stabilizing TAB2. To verify whether the function of microglial OTUB1 in cerebral ischemic injury depends on TAB2, we ectopically expressed FLAG-tagged TAB2 in microglia of *Otub1*^flox^ and *Otub1*^CKO^ mice using adeno-associated virus (AAV) serotype 11 armed with Iba-1 promoter and FLAG-TAB2 sequences (Fig. 6A–C). AAV-mediated overexpression of TAB2 significantly increased the brain infarct volume induced by MCAO in *Otub1*^CKO^ mice (Fig. 6D,E). In addition, TAB2 overexpression aggravated MCAO-induced neurological deficits in *Otub1*^CKO^ mice (Fig. 6F,G). Importantly, TAB2 overexpression blunted the differences in brain infarct volume and neurological deficits between *Otub1*^flox^ and *Otub1*^CKO^ mice (Fig. 6D–G), indicating that microglial OTUB1 enhances cerebral ischemic injury by regulating TAB2.

### Microglial OTUB1 promotes LPS-induced sickness behavior and CNS autoimmune inflammation

**Figure 7:**
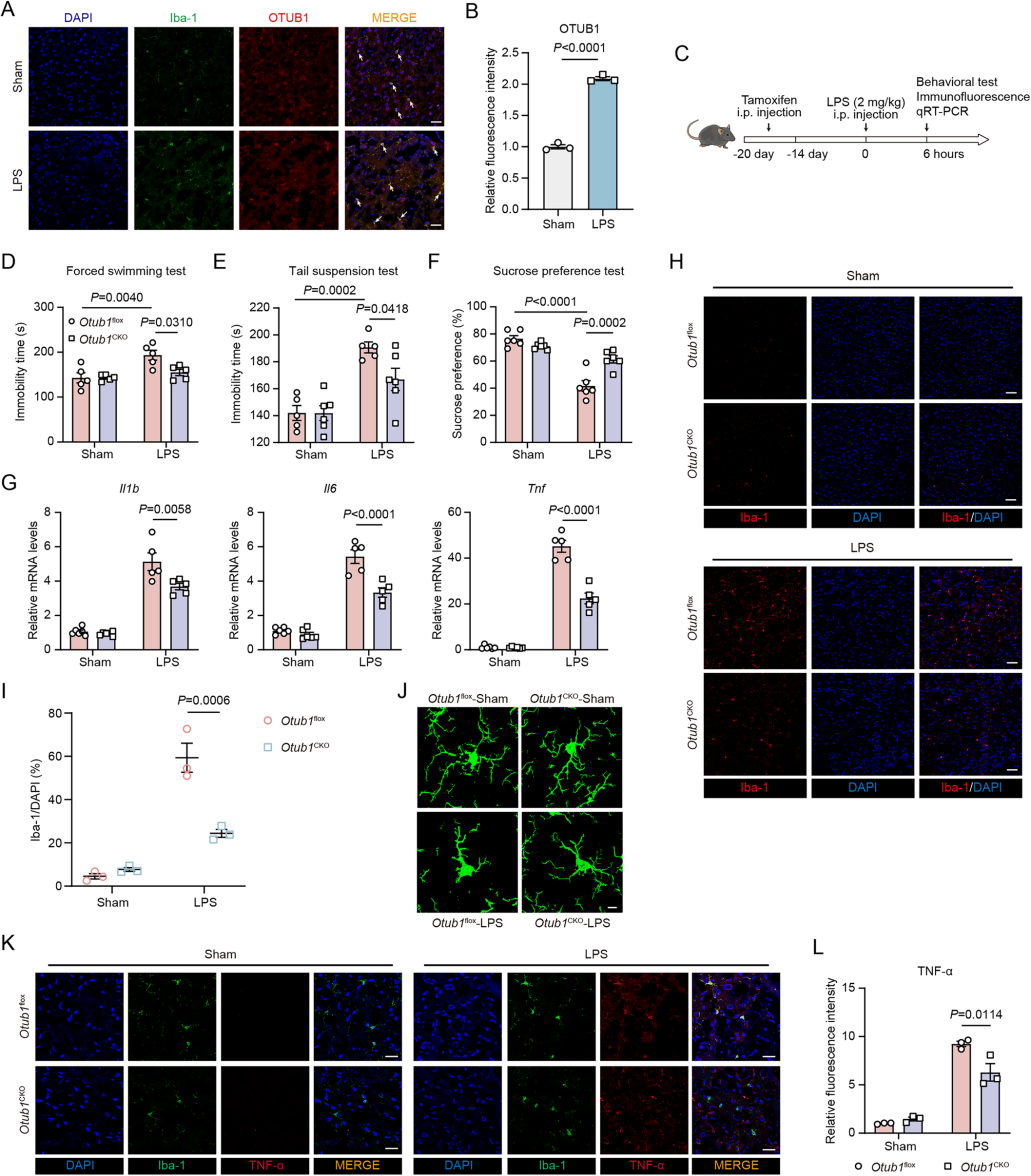
Microglia-specific OTUB1 deficiency ameliorates LPS-induced sickness behavior in mice. (**A**, **B**) Brain sections of mice intraperitoneally administered with PBS (sham) or 2 mg/kg of LPS (LPS) were immunostained with Iba-1 and OTUB1. Representative images (**A**) and quantification of OTUB1 fluorescence intensity in Iba-1^+^ cells (**B**) are shown (*n* = 3, biological replicates). Scale bar = 50 µm. (**C**) Schematic diagram showing the experimental design for LPS treatment. (**D**–**F**) *Otub1*^flox^ and *Otub1*^CKO^ mice treated with PBS or LPS for 6 h were evaluated by forced swim test (**D**), tail suspension test (**E**), and sucrose preference test (**F**) (*n* = 5–6, biological replicates). (**G**) Transcriptional levels of *Il1b*, *Il6*, and *Tnf* in the brain of indicated mice were determined by qRT-PCR (*n* = 5–6, biological replicates). (**H**, **I**) Brain sections of indicated mice were immunostained for Iba-1. Representative images (**H**) and quantification (**I**) are shown (*n* = 3, biological replicates). Scale bar = 50 µm. (**J**) Representative Z-stack images of Iba-1 immunostaining in the ischemic penumbra of indicated mice. Scale bar = 5 µm. (**K**, **L**) Brain sections of indicated mice were immunostained for Iba-1 and TNF-α. Representative images (**K**) and quantification of TNF-α fluorescence intensity in Iba-1^+^ cells (**L**) are shown (*n* = 3, biological replicates). Scale bar = 50 µm. Data in (**B**, **D**–**G**, **I**, **L**) are exhibited as mean ± SEM. Statistics were performed with the two-tailed Student’s *t* test (**B**) or two-way ANOVA test (**D**–**G**, **I**, **L**). Source data are available online for this figure.

Next, we investigated the significance of our findings that OTUB1 promoted TLR-driven microglial activation in other models of neuroinflammatory diseases. Consistent with the in vitro results that LPS induced the upregulation of OTUB1 in microglia (Fig. 2D–F), we found that OTUB1 was upregulated in microglia of mice after LPS administration (Fig. 7A,B). Specific deletion of OTUB1 in microglia significantly ameliorated LPS-induced sickness behaviors in mice, as evidenced by a series of behavioral tests (Fig. 7C–F). Moreover, LPS-induced transcription of *Il1b*, *Il6*, and *Tnf* was significantly attenuated in the brain of *Otub1*^CKO^ mice compared with *Otub1*^flox^ mice (Fig. 7G). Immunofluorescence staining showed that microglial OTUB1 deficiency decreased the quantity and activation of microglia in the brain of LPS-treated mice (Fig. 7H–J). Furthermore, OTUB1 deficiency compromised the ability of microglia to produce TNF-α after LPS stimulation (Fig. 7K,L).

**Figure 8:**
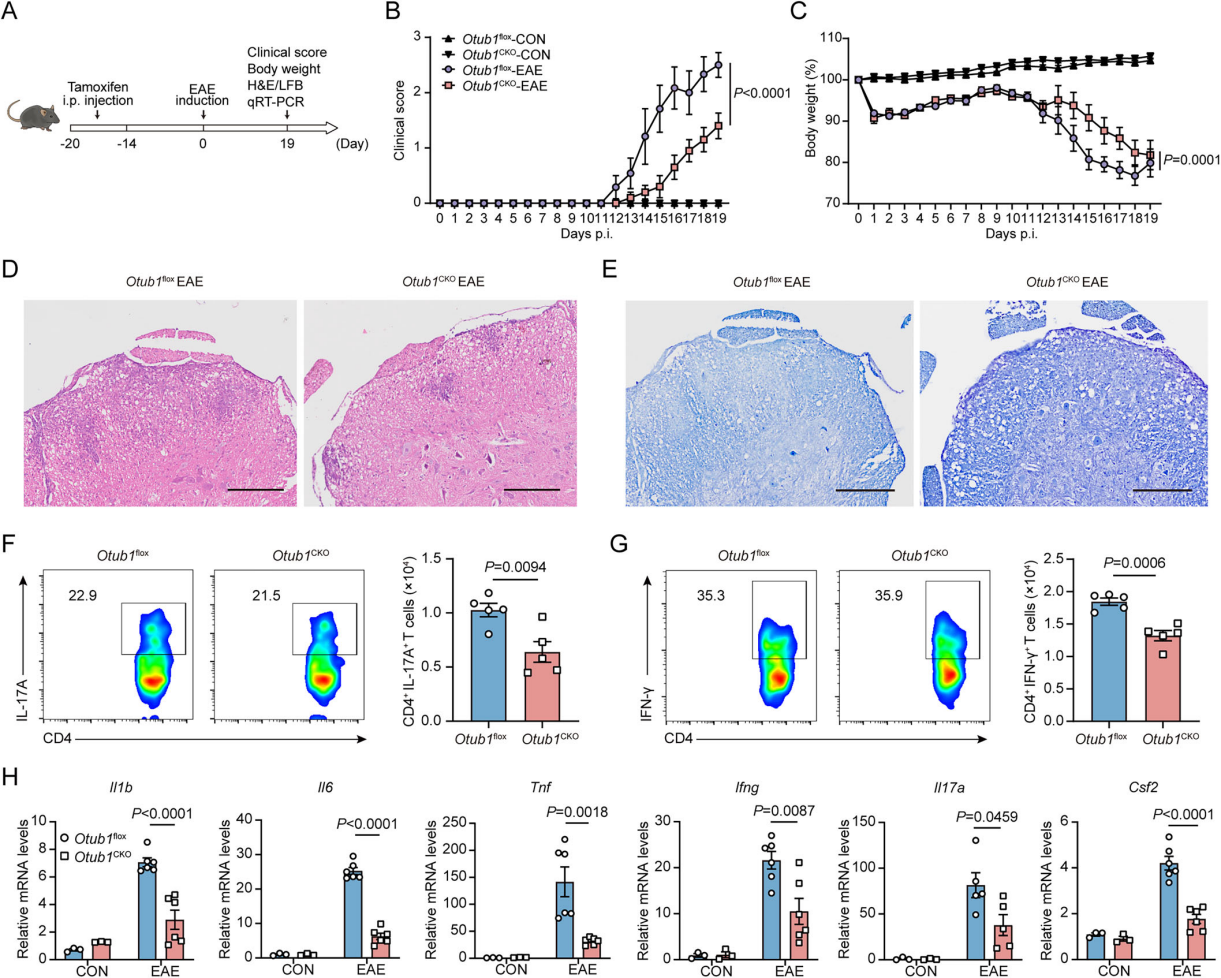
Microglia-specific OTUB1 deficiency ameliorates EAE in mice. (**A**) Schematic diagram showing the timeline for EAE experiments. (**B**, **C**) Clinical scores (**B**) and body weight (**C**) of *Otub1*^flox^ and *Otub1*^CKO^ mice after EAE induction (*n* = 5–6, biological replicates). (**D**, **E**) Spinal cord sections of *Otub1*^flox^ and *Otub1*^CKO^ mice on day 19 post-immunization were analyzed by H&E (**D**) and LFB (**E**) staining. Scale bar = 200 µm. (**F**, **G**) Infiltrating CD4^+^ IL-17^+^ T cells (**F**) and CD4^+^ IFN-γ^+^ T cells (**G**) in the spinal cord of *Otub1*^flox^ and *Otub1*^CKO^ mice on day 19 post-immunization were analyzed by flow cytometry. Representative contour plots (left panels) and absolute numbers (right panels) are shown (*n* = 5, biological replicates). (**H**) Transcriptional levels of *Il1b*, *Il6*, *Tnf*, *Ifng*, *Il17a*, and *Csf2* in the spinal cord of *Otub1*^flox^ and *Otub1*^CKO^ mice on day 19 post-immunization were measured by qRT-PCR (*n* = 3–6, biological replicates). Data in (**B**, **C**, **F**, **G**, **H**) are expressed as mean ± SEM. Statistics were performed with the two-way ANOVA (**B**, **C**, **H**) or two-tailed Student’s *t* (**F**, **G**) test. Source data are available online for this figure.

TLR-induced microglial activation also contributes to CNS autoimmune inflammation (Xiao et al, 2013). Thus, we studied the role of microglial OTUB1 in EAE, a murine model for the human CNS autoimmune disease multiple sclerosis (Fig. 8A). After EAE induction with MOG_35-55_ immunization, *Otub1*^CKO^ mice displayed significantly lower clinical scores and lost significantly less body weight compared with *Otub1*^flox^ mice (Fig. 8B,C), showing that *Otub1*^CKO^ mice are refractory to EAE induction. In comparison to *Otub1*^flox^ mice, *Otub1*^CKO^ mice showed a strong reduction in leukocyte infiltration and demyelination in the spinal cord during EAE (Fig. 8D,E). Although microglia-specific OTUB1 deletion had no discernible effect on the percentage of CD4^+^ IFN-γ^+^ and CD4^+^ IL-17^+^ cells in the spinal cord during EAE, the number of CD4^+^ IFN-γ^+^ and CD4^+^ IL-17^+^ cells in the spinal cord of *Otub1*^CKO^ mice was significantly lower than that in *Otub1*^flox^ mice due to the overall reduction in leukocyte infiltration (Fig. 8F,G). In addition, lower levels of pro-inflammatory cytokines related to CNS autoimmunity were detected in the spinal cord of *Otub1*^CKO^ mice (Fig. 8H). Collectively, these results show that microglial OTUB1 potentiates both LPS-induced sickness behavior and EAE by promoting neuroinflammation, suggesting that OTUB1 serves as an important regulator of microglia-mediated neuroinflammation in TLR-related neuroinflammatory diseases.

### Pharmacological inhibition of OTUB1 ameliorates ischemic brain injury

**Figure 9:**
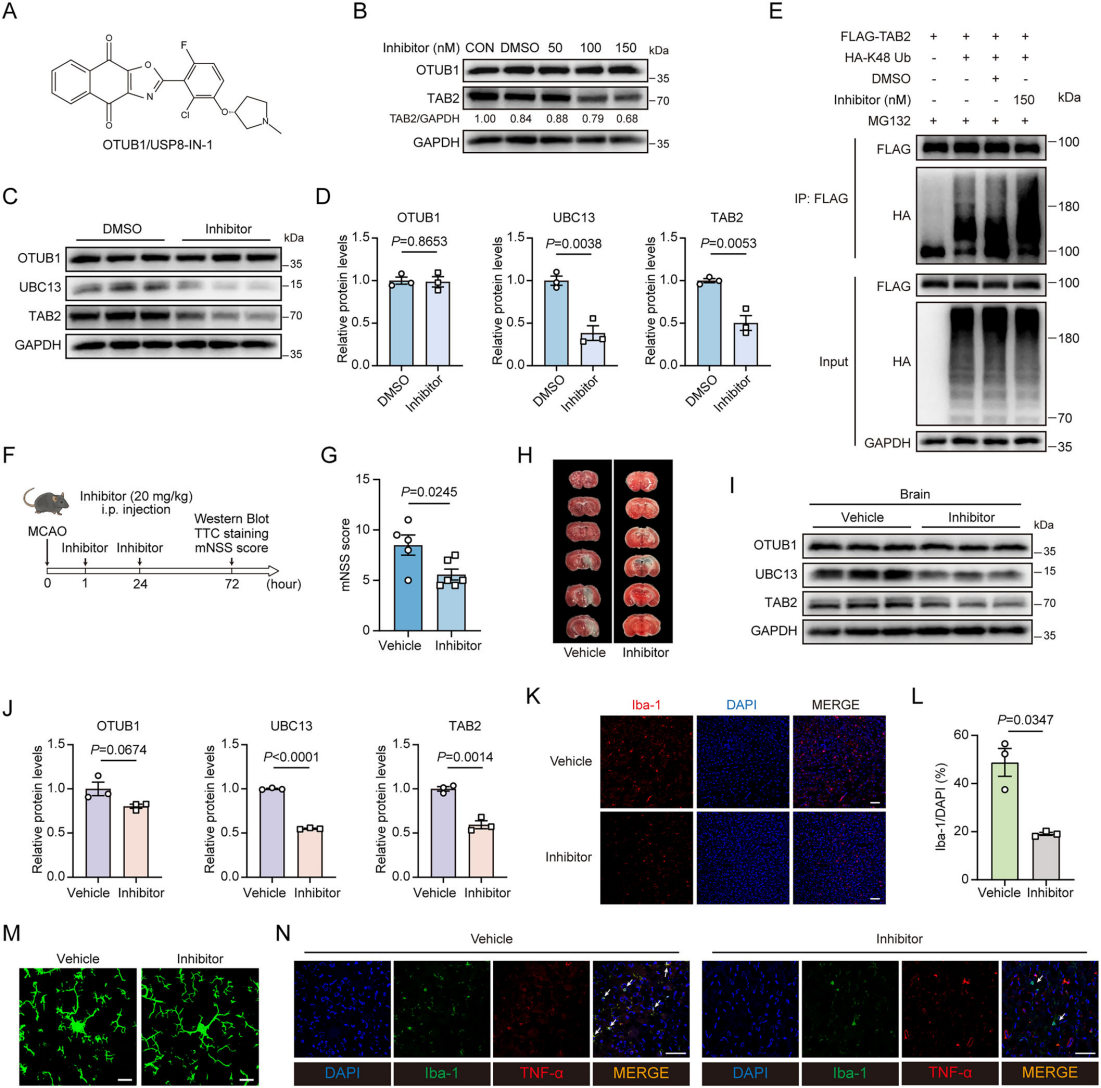
Pharmacological inhibition of OTUB1 attenuates ischemic stroke injury in mice. (**A**) Structural formula of OTUB1/USP8-IN-1. (**B**) Immunoblotting for OTUB1 and TAB2 in BV2 cells treated with indicated concentrations of OTUB1/USP8-IN-1 for 48 h. (**C**, **D**) Immunoblotting for OTUB1, UBC13, and TAB2 in BV2 cells treated with DMSO or OTUB1/USP8-IN-1 (150 nM) for 48 h. Representative immunoblots (**C**) and quantification (**D**) are shown (*n* = 3, biological replicates). (**E**) NIH/3T3 cells transfected with indicated plasmids for 24 h were treated with OTUB1/USP8-IN-1 (150 nM) for 48 h, followed by treatment with MG132 (20 µM) for 6 h. Immunocomplexes harvested with anti-FLAG antibody were analyzed by immunoblotting with indicated antibodies. (**F**) Schematic diagram showing the timeline for inhibitor treatment. (**G**) On day 3 after MCAO, mice were evaluated by the mNSS (*n* = 5–6, biological replicates). (**H**) Representative TTC staining of brain sections on day 3 after MCAO. (**I**, **J**) Immunoblotting for OTUB1, UBC13, and TAB2 in brain lysates on day 3 after MCAO. Representative immunoblots (**I**) and quantification (**J**) are shown (*n* = 3, biological replicates). (**K**, **L**) Brain sections were immunostained for Iba-1 on day 3 after MCAO. Representative images (**K**) and quantification (**L**) are shown (*n* = 3, biological replicates). Scale bar = 50 µm. (**M**) Representative Z-stack images of Iba-1 immunostaining in the ischemic penumbra on day 3 after MCAO. Scale bar = 10 µm. (**N**) Brain sections of indicated mice were immunostained for Iba-1 and TNF-α on day 3 after MCAO. Data in (**D**, **G**, **J**, **L**) are displayed as mean ± SEM. Statistics were performed with the two-tailed Student’s *t* test. Source data are available online for this figure.

The identification that genetic ablation of microglial OTUB1 significantly ameliorated cerebral ischemic injury in mice indicated OTUB1 inhibition as a possible therapeutic approach for ischemic stroke. OTUB1/USP8-IN-1 is a small-molecule compound that specifically inhibits OTUB1 and USP8 (Fig. 9A) (Tan et al, 2022). Treatment with OTUB1/USP8-IN-1 significantly reduced TAB2 and UBC13 protein levels in BV2 cells without affecting cell viability (Fig. 9B–D; Appendix Fig. S6A). Moreover, OTUB1/USP8-IN-1 inhibited USP8 and thereby reduced the protein abundance of its target protein EGFR in BV2 cells (Appendix Fig. S6B,C). In contrast, OTUB1/USP8-IN-1 exerted no inhibitory effect on YOD1 and OTUB2, two DUBs sharing structural similarity with OTUB1, as well as their targets RIPK2 and RIPK3 (Appendix Fig. S6B,C), confirming the specificity of OTUB1/USP8-IN-1 toward OTUB1 and USP8 (Mei et al, 2025; Shen et al, 2024). Furthermore, K48 ubiquitination of TAB2 was increased in the presence of OTUB1/USP8-IN-1 treatment (Fig. 9E), consolidating our previous finding that OTUB1 reduced the K48 ubiquitination of TAB2. Notably, siRNA-mediated downregulation of USP8 significantly reduced EGFR levels but did not reduce the abundance of TAB2 and UBC13 (Appendix Fig. S6D,E), ruling out the possibility that OTUB1/USP8-IN-1 regulates the ubiquitination and stability of TAB2 by inhibiting USP8. Considering that OTUB1/USP8-IN-1 could pass the blood-brain barrier (BBB), we analyzed the efficacy of OTUB1/USP8-IN-1 in treating ischemic stroke injury in mice and found that OTUB1/USP8-IN-1 treatment significantly diminished neurological deficits and cerebral infarction in mice subjected to MCAO (Fig. 9F–H; Appendix Fig. S6F,G). In addition, protein levels of UBC13, TAB2, USP8, and EGFR were significantly reduced in the brain of OTUB1/USP8-IN-1-treated mice (Fig. 9I,J; Appendix Fig. S6H,I). OTUB1/USP8-IN-1 treatment also decreased the number, activation, and cytokine production of microglia in the brain after ischemic insult (Fig. 9K–N). Taken together, these data indicate that pharmacological inhibition of OTUB1 confers protection against ischemic brain injury, which should be largely attributed to the downregulation of microglial activation.

## Discussion

Microglia-mediated neuroinflammation promotes the onset and development of various CNS diseases. In this study, we discovered that microglial activation and neuroinflammation were tightly controlled by the DUB OTUB1. Genetic deletion of microglial OTUB1 significantly ameliorated multiple neuroinflammatory diseases by inhibiting the pro-inflammatory activation of microglia. Although OTUB1 was mainly deleted in microglia in Cx3cr1-Cre^ERT2^ *Otub1*^fl/fl^ mice after tamoxifen treatment, perturbed OTUB1 in peripheral myeloid populations and border-associated macrophages may also affect neuroinflammation. Moreover, pharmacological inhibition of OTUB1 attenuated cerebral ischemic injury in mice, emphasizing the potential of OTUB1 inhibition in the treatment of ischemic stroke.

TLR-mediated signaling is essential for the activation of microglia (Bell et al, 2013; Jadhav and Khatri, 2025; Xiao et al, 2013). In this study, we discovered that TLR-mediated microglial activation was enhanced by OTUB1, which stabilized UBC13 and TAB2 to promote NF-κB and MAPK signaling. Our findings are in conformance with a previous report showing that OTUB1 augments TLR signaling in dendritic cells by stabilizing UBC13 (Mulas et al, 2021). In addition to UBC13, TAB2 was identified in this study as a novel target of OTUB1. Of note, some DUBs modulate the NF-κB pathway by regulating multiple signaling molecules. For instance, A20 inhibits TLR signaling by regulating TRAF6 and NEMO (Boone et al, 2004; Shembade et al, 2010; Skaug et al, 2011). Similarly, the DUB USP25 inhibits TLR-induced signal transduction by deubiquitinating TRAF3, TRAF6, and TAB2 (Li et al, 2023; Liu et al, 2023; Zhong et al, 2013). In contrast to A20 and USP25, which serve as critical shutdown machineries for TLR signaling, OTUB1 is required for the effective activation of this signaling. The simultaneous regulation of UBC13 and TAB2, two essential signaling molecules in TLR signaling, by OTUB1 underscores the importance of OTUB1 in the regulation of TLR signaling.

Upon TLR activation, TAB2 binds to K63-linked polyubiquitin chains on TRAF6, leading to the autophosphorylation-dependent activation of TAK1 and the subsequent activation of downstream NF-κB and MAPK pathways (Kanayama et al, 2004). Due to its critical function in signal transduction, TAB2 protein levels are precisely regulated to ensure the optimal activation of MAPK and NF-κB pathways. Accumulating studies reveal that TAB2 can be degraded through K48 ubiquitination-dependent proteasomal degradation and ubiquitination-independent lysosomal degradation (Zhou et al, 2020). The E3 ligases RBCK1 and RNF99 cause the proteasomal degradation of TAB2 by inducing K48 ubiquitination, whereas K48 deubiquitination of TAB2 by ASB1 reduces its degradation (Hou et al, 2021; Tian et al, 2007; Zhang et al, 2023). On the contrary, TRIM30α and TRIM38 induce the lysosome-dependent degradation of TAB2, thereby negatively regulating NF-κB activation (Hu et al, 2014; Shi et al, 2008). Here, we found that OTUB1 specifically inhibited the proteasomal degradation of TAB2 by reducing its K48 ubiquitination, which is in conformance with previous reports that K48 ubiquitination instigates the proteasomal degradation of TAB2 (Hou et al, 2021; Tian et al, 2007; Zhang et al, 2023). Our research and that of others jointly reveal the complex regulation of TAB2 by ubiquitin-modifying enzymes, highlighting the redundancy and compensatory mechanisms of ubiquitination in orchestrating TAB2 activation.

OTUB1 is a special DUB that possesses not only the canonical deubiquitinating activity but also a unique non-canonical function (Nakada et al, 2010; Wiener et al, 2012). The deubiquitinating activity, exerted by the C91 active site, empowers OTUB1 to cleave K48 polyubiquitin chains on substrates (Balakirev et al, 2003). In addition to this canonical activity, OTUB1 can also impede the ubiquitination of substrates by suppressing the activity of E2s in a non-canonical manner, which is dependent on the N-terminus and the D88 residue (Juang et al, 2012; Sun et al, 2012; Wang et al, 2019; Wu et al, 2021). Here, we discovered that, instead of directly cleaving K48-linked polyubiquitination on TAB2, OTUB1 non-canonically hindered the K48 ubiquitination of TAB2. Moreover, this non-canonical regulation of TAB2 by OTUB1 was mediated by the N-terminus, which is also required for the non-canonical function of OTUB1 in regulating SOCS1 and SMAD3 (Herhaus et al, 2013; Wang et al, 2019).

Activated NF-κB and MAPK signaling drives the generation of pro-inflammatory cytokines, particularly TNF-α, in microglia. In addition to amplifying inflammatory responses in various cell types including microglia, TNF-α induces various forms of cell death, including necroptosis and apoptosis (van Loo and Bertrand, 2023; Yuan and Ofengeim, 2024). Considering that necroptosis and apoptosis are two principal mechanisms regulating neuronal death after cerebral ischemia, the magnitude of TNF-α production by microglia dictates the severity of neurological damage in ischemic stroke (Mei et al, 2025; Naito et al, 2020; Zhu et al, 2025). In this study, we observed that the improved neurological function of mice was correlated with reduced TNF-α production in microglia upon genetic deletion or pharmacological inhibition of OTUB1. These results also underscore the significance of inflammation regulation in the treatment of neuroinflammatory diseases such as cerebral ischemic stroke.

Inhibition of OTUB1 with the small-molecule inhibitor OTUB1/USP8-IN-1 decreased microglial activation, reduced cerebral infarct size, and improved neurological functions after ischemic brain insult. However, we have to point out that OTUB1/USP8-IN-1 is a non-specific inhibitor for OTUB1, as it inhibits both OTUB1 and USP8 (Tan et al, 2022). The lack of specific OTUB1 inhibitors restricts the validation of OTUB1 as a therapeutic target for neuroinflammatory diseases such as ischemic stroke, and this is a limitation of our study. Given that USP8 plays a beneficial role in cerebral ischemic injury and that USP8 inhibits TLR-mediated microglial activation (Zhang et al, 2017; Zhao et al, 2020), the therapeutic effect of OTUB1/USP8-IN-1 on ischemic stroke should be attributed solely to the inhibition of OTUB1. In addition, downregulation of USP8 with siRNA had no influence on UBC13 and TAB2 expression, further corroborating that OTUB1/USP8-IN-1-mediated downregulation of UBC13 and TAB2 is due to OTUB1 inhibition. In the future, the research and development of specific and potent inhibitors targeting the non-canonical function of OTUB1 may be beneficial for the treatment of ischemic stroke.

In summary, this study establishes OTUB1 as a mediator of microglial activation under neuroinflammatory conditions. Given that signaling pathways induced by most TLRs converge on a signaling complex consisting of TRAF6, UBC13, TAB2, and TAK1, the tight regulation of UBC13 and TAB2 by OTUB1 potentiates TLR-induced signal transduction. The development of specific inhibitors for OTUB1 will be beneficial for the further validation of OTUB1 as a therapeutic target for neuroinflammatory diseases.

## Methods

**Table:** Reagents and tools table

Reagent/resource	Reference or source	Identifier or catalog number
**Experimental models**		
C57BL/6 (*M. musculus*)	Cyagen Biosciences	N/A
*Cx3cr1-Cre*^*ERT2*^ (*M. musculus*)	Cyagen Biosciences	N/A
BV2 cells	National Collection of Authenticated Cell Cultures	N/A
NIH/3T3 cells	National Collection of Authenticated Cell Cultures	N/A
*Otub1*^−/−^ BV2 cells	This study	N/A
**Plasmids/vectors**		
GFP-Vector	OriGene	N/A
GFP-OTUB1	OriGene	N/A
GFP-OTUB1-86-271	OriGene	N/A
GFP-OTUB1-ΔN	OriGene	N/A
GFP-OTUB1-D88A	OriGene	N/A
GFP-OTUB1-C91S	OriGene	N/A
FLAG-Vector	GeneChem	N/A
FLAG-TAB2	GeneChem	N/A
HA-Ub	GeneChem	N/A
HA-K48 Ub	GeneChem	N/A
HA-K63 Ub	GeneChem	N/A
**Oligonucleotides and other sequence-based reagents**		
Primers for quantitative Real-Time PCR (qRT-PCR)	This study	Appendix Table S1
**Antibodies (dilution)**		
NeuN WB1:1000, IF1:200	Abcam/Proteintech	ab177487/66836-1-Ig
Iba-1 IF1:200	Cell Signaling Technology	17198S
K48-linkage Specific Polyubiquitin WB1:1000	Cell Signaling Technology	8081S
p38 WB1:1000	Cell Signaling Technology	8690S
p65 WB1:1000	Cell Signaling Technology	8242S
p-Erk1/2 WB1:2000	Cell Signaling Technology	4370S
p-JNK WB1:1000	Cell Signaling Technology	4668S
p-p38 WB1:1000	Cell Signaling Technology	9211S
p-p65 WB1:1000	Cell Signaling Technology	3033S
RIPK2 WB1:1000	Cell Signaling Technology	4142S
RIPK3 WB1:1000	Cell Signaling Technology	95702S
TAK1 WB1:1000	Cell Signaling Technology	5206S
UBE2N/Ubc13 WB1:1000	Cell Signaling Technology	4919S
OTUB1 WB1:1000, IF1:200	Novus Biologicals	NBP1-49934
OTUB2 WB1:1000	Novus Biologicals	NBP2-03223
FLAG tag WB1:1000	Proteintech	20543-1-AP
GAPDH WB1:1000	Proteintech	60004-1-Ig
GFP tag WB1:1000	Proteintech	50430-2AP
HA tag WB1:1000	Proteintech	51064-2-AP
LAMP1 IF1:200	Proteintech	21997-1-AP
PSD95 WB1:1000	Proteintech	20665-1-AP
PSMD7 IF1:200	Proteintech	16034-1-AP
TAB2 WB1:1000	Proteintech	14410-1-AP
USP8 WB1:1000	Proteintech	27791-1-AP
YOD1 WB1:1000	Proteintech	25370-1-AP
**Chemicals, enzymes and other reagents**		
Phosphatase Inhibitor Cocktail	APE×BIO	K1015
Protease Inhibitor Cocktail	APE×BIO	K4002
BeyoMag^TM^ Protein A + G Magnetic Beads	Beyotime	P2108
Quick Start^TM^ Bradford Protein Assay Kit	BIO-RAD	5000201
Protein Extraction Reagents	Boster Bio	AR0101/AR0103
5× DualColor Protein Loading Buffer	FUDE Biological Technology	FD006
DMEM medium	Gibco	11965118
Glucose-Free DMEM	Gibco	11966025
Opti-MEM^TM^	Gibco	31985070
0.25% Trypsin	Gibco	25200072
Chloroquine	MedChemExpress	HY-17589A
Corn oil	MedChemExpress	HY-Y1888
MG132	MedChemExpress	HY-13259
OTUB1/USP8-IN-1	MedChemExpress	HY-151563
Cycloheximide	Merck	239763-M
Cell Counting Kit-8	New Cell & Molecular Biotech	C6005
Ncm ECL Ultra	New Cell & Molecular Biotech	P10300
2, 3, 5-triphenyltetrazolium chloride (TTC)	Sigma-Aldrich	T8877
5% BSA Blocking Buffer	Solarbio	SW3015
DMSO	Solarbio	D8371
Hematoxylin-Eosin (H&E)	Solarbio	G1120
Luxol Fast Blue Myelin Stain Kit (Cresyl Violet Method)	Solarbio	G3245
Paraformaldehyde	Solarbio	P1110
Penicillin-Streptomycin Liquid	Solarbio	P1400
0.5% Triton X-100	Solarbio	T8200
Brain Slicing Mold	RWD life science	68707
Isoflurane	RWD life science	R510-22-10
MCAO sutures	RWD life science	MSMC21B120PK50/MSMC23B120PK50
PrimeScript^TM^ RT reagent Kit	Takara	RR037A
TB Green^®^ Premix Ex Taq^TM^ II	Takara	RR820A
Lipofectamine 3000	Thermo Fisher Scientific	L3000015
TRIzol Reagent	Thermo Fisher Scientific	15596026CN
Fetal Bovine Serum	Vazyme	F101-01
**Software**		
MultiQuant^TM^ 3.0.3 software	SCIEX	N/A
GraphPad Prism 8	GraphPad	N/A
ImageJ software	NIH Image	N/A
SPSSAU	www.spssau.com	N/A
GraphBBB	www.graphbbb.com	N/A
Evolution Capt	Vilber	N/A
**Other**		
Hypoxic chamber	Billups-Rothenberg	MIC-101
Panlab Rotarod	Harvard Apparatus	76-0770
Forma^TM^ 3 cell incubator	Thermo Fisher Scientific	4110
Liquid chromatography-triple quadrupole mass spectrometer	SCIEX	Qtrap6500 + /Triple Quad^TM^ 4500MD
Multiskan SkyHigh Microplate Spectrophotometer	Thermo Fisher Scientific	A51119700DPC
QuantStudio^TM^ 5 Real-Time PCR System	Thermo Fisher Scientific	Quant Studio^TM^ 5
FV3000 Confocal Laser Scanning Microscope	Olympus	Evident/FV3000
Leica laser scanning confocal microscope	Leica	STELLARIS 5
Fusion FX.EDGE system	Vilber	N/A

### Experimental animals

*Otub1*^fl/fl^ (*Otub1*^flox^) mice were generated as described before (Mulas et al, 2021; Wang et al, 2019). Cx3cr1-Cre^ERT2^ mice were provided by Cyagen. *Otub1*^fl/fl^ mice were bred with Cx3cr1-Cre^ERT2^ mice to produce Cx3cr1-Cre^ERT2^ *Otub1*^fl/fl^ (*Otub1*^CKO^) mice. Genotyping was carried out by PCR with the following primers: OTUB1.1 s: 5’-GAGGTAGGTGATGCTCAGGTG-3’, OTUB1.1 as: 5’-CTTACTGGGAAAGAAGCTTGC-3’ (wildtype: 216 bp, flox: 376 bp); Cx3crlCreER-Common (12266) 5’-AAGACTCACGTGGACCTGCT-3’, Cx3crlCreER-Mutant Reverse (14314) 5’-CGGTTATTCAACTTGCACCA-3’, Cx3crlCreER-WT Reverse (16221) 5’-AGGATGTTGACTTCCGAGTTG-3’ (positive: 300 bp, negative: 695 bp). PCR primers were obtained from Sangon Biotech. To induce the deletion of OTUB1, 2-month-old mice were daily injected with 100 mg/kg of tamoxifen for 7 days. Mice were kept in specific pathogen-free (SPF) environment, with unrestricted access to food and water. Animals were randomly assigned to control and experimental groups, and blinding was applied for animal experiments. The animal experiments were carried out in conformity with regulations and approved by the Animal Policy and Welfare Committee of Wenzhou Medical University (Approval numbers: wydw2025-0208, wydw2025-0470, wydw2025-0471).

### Cell culture

BV2 and NIH/3T3 cells were cultured in Dulbecco’s Modified Eagle Medium (DMEM) with 1% penicillin/streptomycin and 10% fetal bovine serum (FBS). The CRISPR/Cas9 technology (*Otub1*-gRNA: AGAATCCTCTGGTGTCAGAG) was utilized to produce *Otub1*^−/−^ cell lines as described before (Zhao et al, 2023). To obtain primary microglia, mixed glial cells were harvested from the brain of newborn mice and cultured in DMEM with 1% penicillin/streptomycin and 10% FBS. After 10 days of culture, astrocytes were removed through digestion with trypsin diluted in DMEM. After washing with PBS, the adherent primary microglia were further cultured in DMEM with 1% penicillin/streptomycin and 10% FBS.

### MCAO surgery

Unilateral focal cerebral ischemia was established through the MCAO surgery. During the surgery, mice were anesthetized with 1.5% isoflurane, and a heating pad was utilized to maintain their body temperature at 37.0 ± 0.5 °C. Under a stereo microscope, a midline cervical incision was made, exposing the internal carotid artery (ICA), the external carotid artery (ECA), and the left common carotid artery (CCA). A small incision was made on the ECA using a pair of ophthalmic scissors. Then, a silicone-coated nylon filament was carefully placed into the ICA through the incision. After that, the filament was advanced along the ICA until its silicone tip reached the middle cerebral artery (MCA). A laser Doppler flowmeter was applied to monitor cerebral blood flow (CBF). MCA was properly occluded when CBF dropped to below 25% of the baseline value. After occlusion, the filament was pulled out, restoring CBF to over 70% of baseline. Mice were excluded if their CBF was not reduced to below 25% of baseline or if they showed no neurological deficits after recovery from anesthesia. Mice in the sham operation group experienced similar surgical procedures as mice in the MCAO group, except for the filament insertion. After the surgery, the mice were kept in a heated incubator for recovery.

### Measurement of cerebral infarct volume

Mice were euthanized and then subjected to intracardiac perfusion with saline water. After that, the brains were promptly isolated and cut into 1-mm-thick coronal slices with the aid of a mold. The slices were incubated in 1% 2,3,5-triphenyltetrazolium chloride (TTC) at 37 °C in the dark for 10 min, and then fixed in 4% paraformaldehyde (PFA) for 5 min. Images were taken using a digital camera, and the cerebral infarct size was quantified using the ImageJ software (NIH).

### EAE induction and assessment

EAE was induced in female mice by active immunization with the MOG_35-55_ peptide. Briefly, each mouse was subcutaneously injected with 200 µg of MOG_35-55_ peptide mixed in complete Freund’s adjuvant. On days 0 and 2 post-immunization, each mouse was intraperitoneally injected with 200 ng of pertussis toxin. Clinical signs of the immunized mice were scored daily by two independent investigators in a blinded manner according to a scale ranging from 0 to 5: 0, no abnormality; 0.5, partial tail weakness; 1, limp tail; 1.5, slowing of righting; 2, hindlimb weakness; 2.5, dragging of hindlimbs; 3, hindlimb paralysis; 3.5, slight forelimb weakness; 4, severe forelimb weakness; 5, moribund or dead. In addition, body weight was also monitored daily.

### Behavioral assessment

The mNSS, corner turning test, and rotarod test were applied to evaluate the neurological function of mice after MCAO. The mice were trained for these tests for 3 days before the MCAO surgery. Mice were intraperitoneally administered 2 mg/kg of LPS to induce sickness behavior. Six hours after LPS administration, mice were assessed by the tail suspension test, sucrose preference test, and forced swim test. Behavioral tests were conducted by independent investigators who did not know the identity of the mice.

### Immunoblot analysis

Cultured cells or animal tissue were lysed in lysis buffer for protein extraction. Protein concentration was measured using Quick Start Bradford 1× Dye Reagent, and then the samples were denatured in 5× loading buffer at 100 °C for 10 min. After sodium dodecyl sulfate-polyacrylamide gel electrophoresis (SDS-PAGE), the samples were transferred to polyvinylidene fluoride (PVDF) membranes. The membranes were incubated at room temperature with 5% skim milk for 2 h, and then incubated at 4 °C with primary antibodies overnight. Thereafter, the membranes were incubated at room temperature with secondary antibodies conjugated with horseradish peroxidase (HRP) for 1 h. Subsequently, the membranes were developed with NcmECL Ultra. Images were acquired using the Fusion FX.EDGE system (Vilber), and band intensity was quantified using the ImageJ software.

### Plasmid transfection

Plasmids encoding GFP-OTUB1, GFP-OTUB1-86-271, GFP-OTUB1-ΔN, GFP-OTUB1-D88A, GFP-OTUB1-C91S, and GFP were described before (Zhao et al, 2023). FLAG-TAB2 plasmids were constructed by GeneChem. Plasmids encoding HA-tagged ubiquitin and its mutants were obtained from Miaoling Plasmid Platform. Plasmids were transfected into NIH/3T3 cells using Lipomaster 3000.

### Stereotactic injection

AAV11 viral vectors encoding FLAG-tagged TAB2 and control viral vectors were injected into mice via intracerebroventricular injection. The injection coordinates were referenced to the bregma point: anteroposterior −0.22 mm, mediolateral +1.00 mm, and dorsalvental −2.20 mm. In total, 1 μL of either AAV-FLAG-CON (1.0E + 12 v.g) or AAV11-Iba-1-FLAG-TAB2 (1.0E + 12 v.g) was administered to each mouse at an injection rate of 200 nL per minute.

### Flow cytometry

Infiltrating leukocytes were isolated from mouse spinal cords using Percoll gradient centrifugation. For intracellular cytokine staining, the isolated cells were stimulated with PMA (50 ng/mL), ionomycin (500 ng/mL), and brefeldin A (1 μg/mL) for 4 h at 37 °C in a humidified incubator with 5% CO_2_. After surface staining, the cells were fixed and permeabilized with Fixation/Permeabilization solution and Perm/Wash buffer. The cells were then incubated with monoclonal antibodies for 20 min for intracellular staining. Finally, data were acquired on a Beckman CytoFLEX LX flow cytometer and analyzed using FlowJo software.

### RNA interference

The siRNA targeting *Otub1* (sense: GCGACUCCGAAGGUGUUAATT) was synthesized by RiboBio, and the siRNA targeting *Usp8* (sense: CGGAACCUCAAUCCUGUAUUUdTdT) was synthesized by Hippo Biotechnology. Cells were transfected with siRNA using Lipomaster 2000. Forty-eight hours after transfection, the cells were subjected to further analysis.

### RNA extraction and qRT-PCR

The FreeZol Reagent was used to isolate RNA, which was then reverse-transcribed into cDNA with the HiScript IV All-in-One Ultra RT SuperMix for qPCR. Subsequently, TB Green^®^ Premix Ex Taq^TM^ (Tli RNase H Plus) and corresponding primers were used to perform qRT-PCR on a QuantStudio^TM^ 5 Real-Time PCR System. Primers were listed in Appendix Table S1.

### Administration of inhibitors

Cells were treated with OTUB1/USP8-IN-1 that was dissolved in 1% DMSO. For in vivo administration, mice received intraperitoneal injections of OTUB1/USP8-IN-1 dissolved in corn oil. Mice in the control group were administered equal volumes of solvent.

### OGD treatment

Glucose-free DMEM without FBS was added to BV2 cells. Subsequently, the cells were incubated in a hypoxia chamber filled with 5% CO_2_ and 95% N_2_ at 37 °C for 6 h. Thereafter, the culture medium was replaced with normal DMEM supplemented with 10% FBS. The cultures were further incubated in a standard cell incubator with 5% CO_2_ and 95% air for 12 h.

### Cell viability assay

CCK-8 reagent was diluted in DMEM at a ratio of 1:10. Subsequently, 100 μL of the working solution was added to each well of the 96-well plate. Then, the plate was incubated in a cell incubator at 37 °C for 1 h. After that, absorbance at 450 nm was recorded using a Multiskan SkyHigh Microplate Spectrophotometer.

### Immunofluorescence staining

Mice were euthanized and then intracardially perfused with saline water. Brains were isolated and fixed in 4% PFA for 48 h, followed by dehydration in 20% and 30% sucrose for 2 days. After that, frozen brains embedded in Tissue-Tek^®^ O.C.T. Compound were sectioned into 5 μm or 20 μm slices. Cells were cultured in glass-bottom dishes and then fixed with 4% PFA for 10 min, followed by permeabilization with 0.5% Triton X-100 for 10 min. The samples were subjected to blocking with 5% BSA at 37 °C for 60 min. Subsequently, the samples were incubated at 4 °C with primary antibodies overnight, followed by a 1-h incubation with fluorescence-conjugated secondary antibodies at 37 °C in the dark. Finally, nuclear staining was performed using DAPI. Fluorescence images were taken using a STELLARIS 5 Confocal Microscope (Leica) and analyzed with the ImageJ software.

### Immunoprecipitation

Protein samples were generated as described in “Immunoblot analysis”. The samples were incubated with BeyoMag^TM^ Protein A + G Magnetic Beads on a rotator at 4 °C for 2 h. Then, the pre-clearing magnetic beads with non-specific binding proteins were removed by centrifugation. The collected supernatant was incubated with corresponding antibodies on a rotator at 4 °C overnight. Thereafter, magnetic beads were added to the samples, which were then further incubated with rotation at 4 °C for 3 h. The magnetic beads were rinsed five times with ice-cold PBS and then harvested for subsequent experimental analysis.

### In vitro ubiquitination and deubiquitination assays

For the in vitro ubiquitination assay, GFP-OTUB1, GFP-OTUB1-ΔN, GFP, and FLAG-TAB2 proteins were purified from NIH/3T3 cells transfected with corresponding plasmids through immunoprecipitation. Then, FLAG-TAB2 was incubated separately with the same amount of GFP, GFP-OTUB1, or GFP-OTUB1-ΔN proteins in the presence of HA-K48 Ub, 5 mM ATP, and rabbit reticulocyte lysate in the ubiquitination buffer (100 mM NaCl, 50 mM Tris-HCl, 1 mM DTT, and 2.5 mM MgCl_2_) at 37 °C for 2 h. For the in vitro deubiquitination assay, K48-ubiquitinated TAB2 was purified from NIH/3T3 cells transfected with FLAG-TAB2 and HA-K48 Ub plasmids through immunoprecipitation using the anti-FLAG antibody. After washing with PBS, the beads were then washed with the deubiquitination buffer (50 mM Tris-HCl, 5 mM MgCl₂, 5% glycerol, 2 mM ATP, and 2 mM DTT). Subsequently, K48-ubiquitinated TAB2 was incubated with the same amount of GFP-OTUB1, GFP-OTUB1-ΔN, or GFP proteins in the deubiquitination buffer at 37 °C for 2 h. Finally, the samples were examined with immunoblotting.

### Quantification and statistical analysis

Quantitative data were exhibited as mean ± standard error of the mean (SEM) unless otherwise mentioned. In each experiment, quantification data were derived from at least three independent biological replicates, with the exact number of samples described in figure legends. The GraphPad Prism 8 software (GraphPad) was applied to complete the statistical analysis. Statistical significance was determined using the two-tailed Student’s *t* test, one-way Analysis of Variance (ANOVA) test, two-way ANOVA test, or Mann–Whitney *U* test. Exact statistical tests, power, and *P* values were displayed in Appendix Tables S2–S13. *P* < 0.05 indicated statistical significance.

## Supplementary information

Appendix

Peer Review File

Source data Fig. 1

Source data Fig. 2

Source data Fig. 3

Source data Fig. 4

Source data Fig. 5

Source data Fig. 6

Source data Fig. 7

Source data Fig. 8

Source data Fig. 9

Appendix Figure Source Data

## Data Availability

This study contains no data deposited in external repositories. The source data of this paper are collected in the following database record: biostudies:S-SCDT-10_1038-S44321-026-00473-x.
