# Supplementary material for: OTUB1 non-canonically inhibits TAB2 ubiquitination to govern microglia-mediated neuroinflammation
Source: EMBO Mol Med. 2026 Jul 2;18(8):3137–56. doi: 10.1038/s44321-026-00473-x (PMC13470327; doi:10.1038/s44321-026-00473-x)
Supplement: Supplementary file 1 [file 44321_2026_473_MOESM1_ESM.pdf]

Appendix for

OTUB1 non-canonically inhibits TAB2 ubiquitination to govern microglia-mediated neuroinflammation

Table of contents

Table of contents 1
Appendix Figure S1 3
Appendix Figure S2 4
Appendix Figure S3 5
Appendix Figure S4 6
Appendix Figure S5 6
Appendix Figure S6 7
Appendix Table S1 Primers for qRT-PCR 9
Statistics Tables 10
Appendix Table S2 10
Appendix Table S3 11
Appendix Table S4 11
Appendix Table S5 12
Appendix Table S6 12
Appendix Table S7 12
Appendix Table S8 13
Appendix Table S9 13

Appendix Table S10 13
Appendix Table S11 14
Appendix Table S12 14
Appendix Table S13 14

Appendix Figure S1

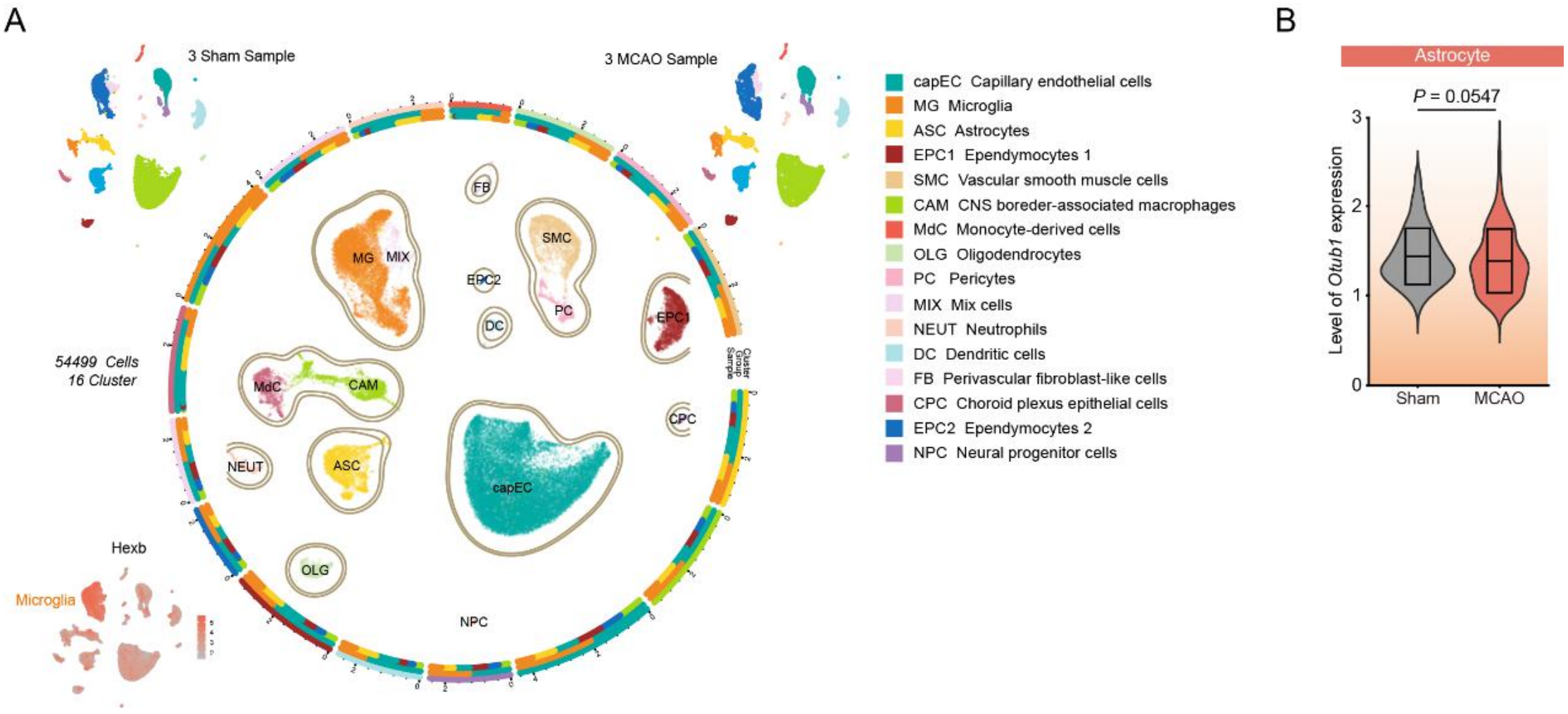

Appendix Figure S1. Transcriptional landscape in mice suffering from ischemic stroke. (A) UMAP plot presenting the results of single-cell sequencing of brain tissues from control mice and mice with ischemic stroke. (B) Violin plot showing the expression levels of *Otub1* in astrocytes of control mice (n=4876, biological replicates) and mice with ischemic stroke (n=6102, biological replicates) based on GSE 174574. Data in (B) are displayed as mean ± SD. Statistics were performed with the Mann-Whitney U test.

Appendix Figure S2

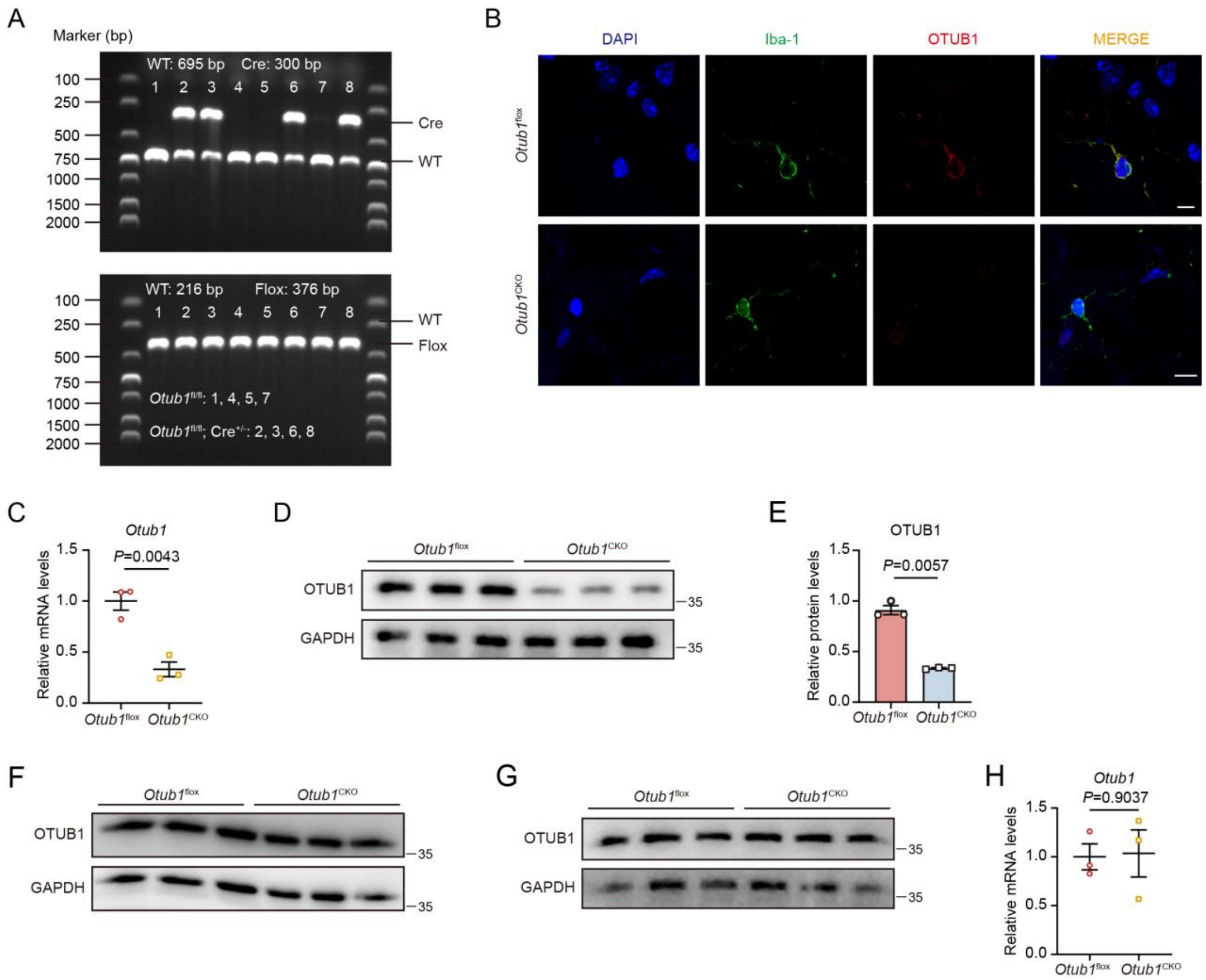

Appendix Figure S2. Genotyping and validation of *Otub1*^{CKO} mice. (A) Genotyping of *Otub1*^{flox} and *Otub1*^{CKO} mice was carried out by PCR. (B) On day 14 after tamoxifen injection, brain sections of *Otub1*^{flox} and *Otub1*^{CKO} mice were immunostained for Iba-1 and OTUB1. Scale bar = 10 μm. (C) On day 14 after tamoxifen injection, *Otub1* mRNA levels in microglia sorted from the brain of *Otub1*^{flox} and *Otub1*^{CKO} mice were determined by qRT-PCR (n = 3, biological replicates). (D-E) Primary microglia isolated from newborn *Otub1*^{flox} and *Otub1*^{CKO} mice were treated with 4-hydroxytamoxifen (1μM), followed by Western blot analysis. Representative immunoblots (D) and quantification (E) are shown (n = 3, biological replicates). (F) On day 14 after tamoxifen injection, OTUB1 expression in peritoneal macrophages of *Otub1*^{flox} and

Otub1^{CKO} mice was analyzed by Western blot. (G-H) On day 14 after tamoxifen injection, bone marrow-derived macrophages were generated using bone marrow from *Otub1*^{flox} and *Otub1*^{CKO} mice. OTUB1 expression was analyzed by Western blot. Representative immunoblots (G) and quantification (H) are shown (n = 3, biological replicates). Data in (C, E, H) are displayed as mean ± SEM. Statistics were performed with the two-tailed Student's t test.

Appendix Figure S3

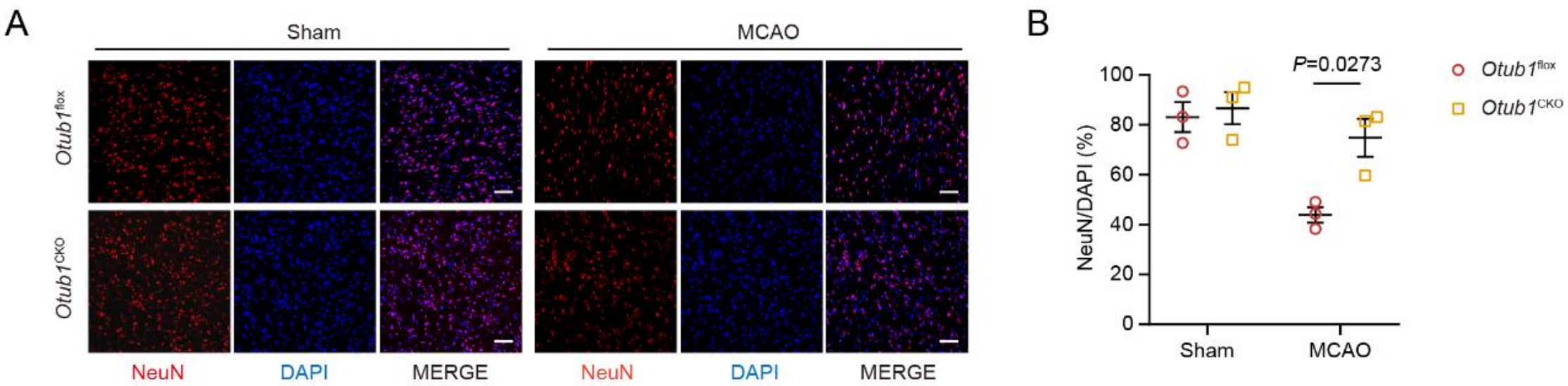

Appendix Figure S3. Microglia-specific OTUB1 deficiency attenuates MCAO-induced neuronal loss. (A-B) On day 2 after MCAO, brain sections of the indicated mice were immunostained for NeuN. Representative images (A) and quantification (B) are shown (n = 3, biological replicates). Scale bar = 50 µm. Data in (B) show mean ± SEM and were analyzed with the two-way ANOVA test.

Appendix Figure S4

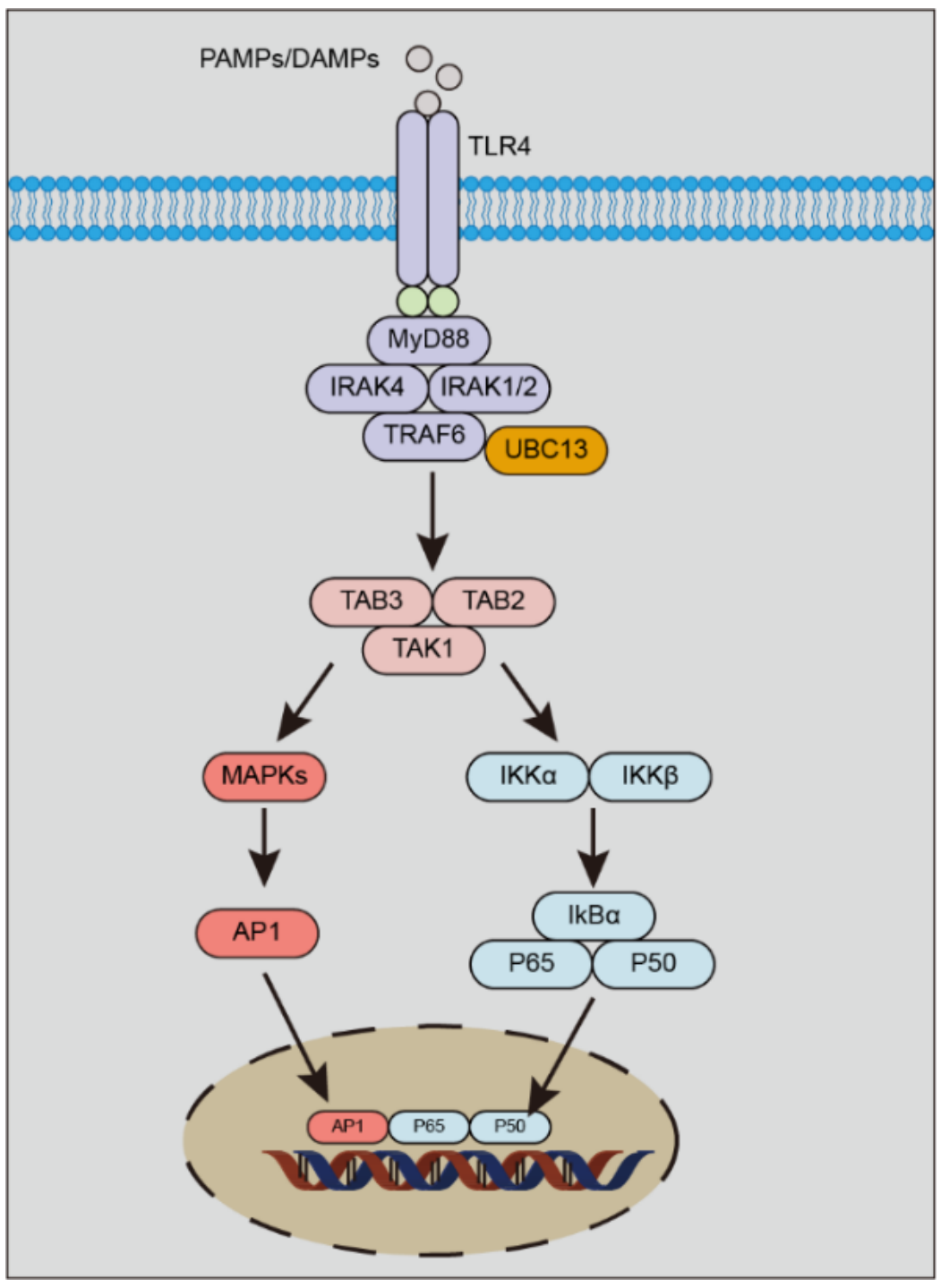

Appendix Figure S4. TLR4-induced signaling pathways.

Appendix Figure S5

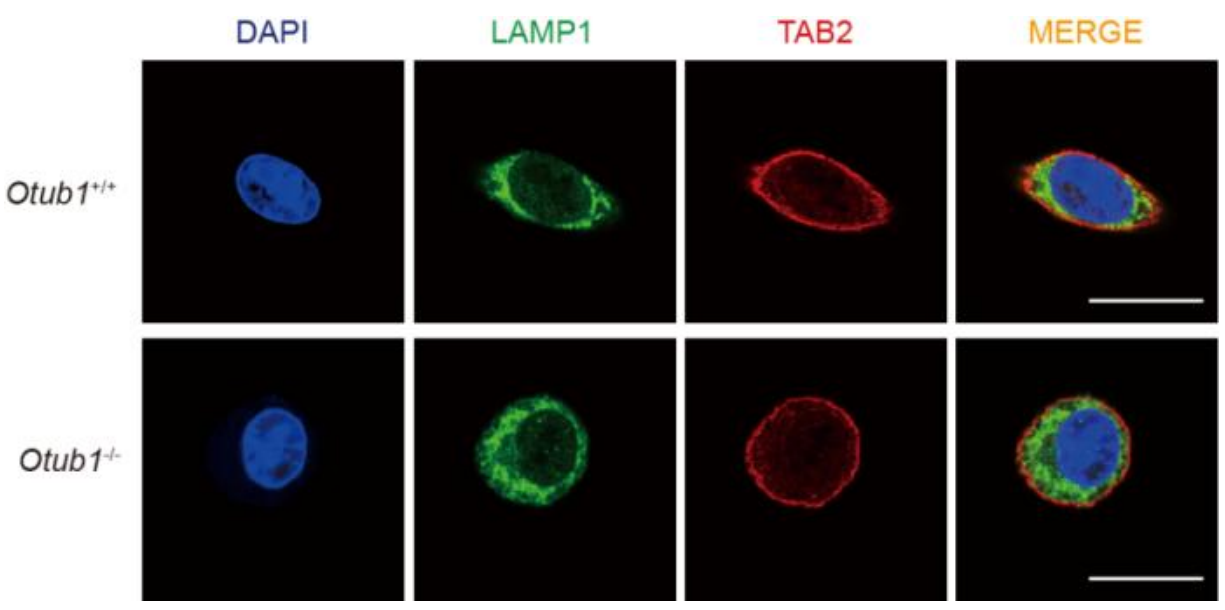

Appendix Figure S5. OTUB1 does not affect the co-localization of TAB2 and LAMP1. *Otub1*$^{+/+}$ and *Otub1*$^{-/-}$ BV2 cells were immunostained for TAB2 and LAMP1. Scale bar = 20 μm.

Appendix Figure S6

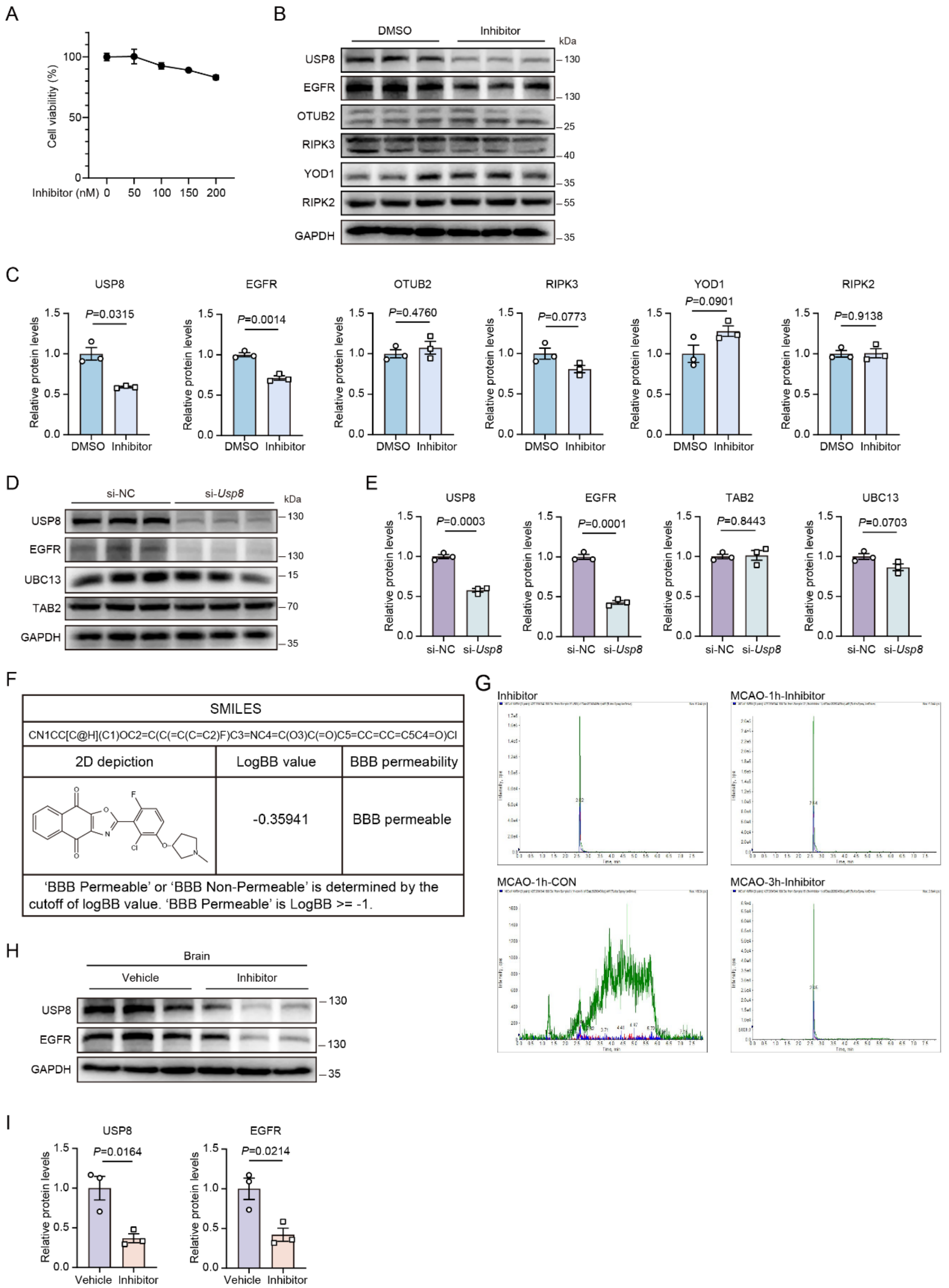

Appendix Figure S6. The effect of OTUB1/USP8-IN-1 on UBC13 and TAB2 is independent of USP8. (A) Cell viability of BV2 cells treated with indicated

concentrations of OTUB1/USP8-IN-1 for 48 h was determined by the CCK8 test (n = 3, biological replicates). (B-C) Immunoblotting for indicated proteins in BV2 cells treated with OTUB1/USP8-IN-1 (150 nM) for 48 h. Representative immunoblots (B) and quantification (C) are shown (n = 3, biological replicates). (D-E) Immunoblotting for indicated proteins in BV2 cells treated with si-NC or si-*Usp8* for 48 h. Representative immunoblots (D) and quantification (E) are shown (n = 3, biological replicates). (F) The BBB permeability of OTUB1/USP8-IN-1 was predicted using an online tool. (G) One hour after MCAO surgery, C57BL/6 mice were intraperitoneally injected with OTUB1/USP8-IN-1 (20 mg/kg). Brain tissues were collected at 1 and 3 hours after administration, and OTUB1/USP8-IN-1 was detected by LC-MS/MS analysis. (H-I) Immunoblotting for USP8 and EGFR in brain lysates on day 3 after MCAO. Representative immunoblots (H) and quantification (I) are shown (n = 3, biological replicates). Data in (C, E, I) represent mean ± SEM and were analyzed with the two-tailed Student's t test.

Appendix Table S1 Primers for qRT-PCR

Reagent/Resource	Reference or source	Identifier or Catalog Number
Il1b reverse primer (M. musculus)	5’-TGAGGCCCAAGGCCACAGGT-3’	N/A
Il1b forward primer (M. musculus)	5’-AAGCCTCGTGCTGTCGGACC-3’	N/A
Il6 reverse primer (M. musculus)	5’-AAGTGCATCATCGTTGTTCATACA-3’	N/A
Il6 forward primer (M. musculus)	5’-ACACATGTTCTCTGGGAAATCGT-3’	N/A
Tnf reverse primer (M. musculus)	5’-ATAGCAAATCGGCTGACGGT-3’	N/A
Tnf forward primer (M. musculus)	5’-ACGTCGTAGCAAACCACCAA-3’	N/A
Ifng reverse primer (M. musculus)	5’-GGGTGGGGAAGAGATTGTCC-3’	N/A
Ifng forward primer (M. musculus)	5’-CCCAGATACAACCCCGCAAT-3’	N/A
Il17a reverse primer (M. musculus)	5’-GCTGACCCCTAAGAAACCCC-3’	N/A
Il17a forward primer (M. musculus)	5’-GAAGCAGTTTGGGACCCCTT-3’	N/A
Csf2 reverse primer (M. musculus)	5’-TAGCTGATAAGGGCCAGGAGA-3’	N/A
Csf2 forward primer	5’-GCTGAGAGGCTGTA	N/A

(M. musculus)	GACCAC-3’	
β-actin reverse primer (M. musculus)	5’-CACAGCTTCTCTTT GATGTCAC-3’	N/A
β-actin forward primer (M. musculus)	5’-CTACCTCATGAAGA TCCTGACC-3’	N/A

Statistics Tables

Appendix Table S2

Figure	Analysis of normality	Statistical method	Power	p value
Fig 1 B (*Otub1*/*Zranb1*/*Usp48*)	No	Mann-Whitney U test	1.000/1.000/1.000	<0.0001/<0.0001/<0.0001
Fig 1 E	Yes	two-way ANOVA followed by the Tukey test	1.000	<0.0001
Fig 1 F	Yes	two-way ANOVA followed by the Tukey test	0.992	0.0001
Fig 1 G	Yes	two-way ANOVA followed by the Tukey test	1.000	<0.0001
Fig 1 I (day3/day7)	Yes	two-way ANOVA followed by the Tukey test	0.988/0.685	0.0003/0.4784
Fig 1 K	Yes	two-way ANOVA followed by the Tukey test	0.791	0.0448
Fig 1 L	Yes	two-way ANOVA followed by the Tukey test	0.831	0.0210
Fig 1 M (*Il1b*/*Il6*/*Tnf*)	Yes	two-way ANOVA followed by the Tukey test	0.752/0.986/0.863	0.0163/0.0002/0.0185
Fig 1 O	Yes	two-way ANOVA followed by the Tukey test	0.946	0.0006

Appendix Table S3

Figure	Analysis of normality	Statistical method	Power	p value
Fig 2 B	Yes	one-way ANOVA followed by the Tukey test	1.000/1.000	0.0011/<0.0001
Fig 2 C	Yes	one-way ANOVA followed by the Tukey test	0.936/0.816	0.0376/0.0272
Fig 2 E	Yes	one-way ANOVA followed by the Tukey test	0.773	0.0127
Fig 2 F	Yes	one-way ANOVA followed by the Tukey test	0.998	0.0222
Fig 2 H (*Il1b*/*Il6*/*Tnf*)	Yes	two-way ANOVA followed by the Tukey test	0.984/0.997/0.988	<0.0001/<0.0001/0.0001
Fig 2 I (IL-1β/IL-6/TNF-α)	Yes	two-way ANOVA followed by the Tukey test	0.971/0.860/0.993	<0.0001/<0.0001/0.0009
Fig 2 J (*Il1b*/*Il6*/*Tnf*)	Yes	two-way ANOVA followed by the Tukey test	0.750/0.576/0.949	0.0097/0.0334/0.0010
Fig 2 K (IL-1β/IL-6/TNF-α)	Yes	two-way ANOVA followed by the Tukey test	0.815/0.895/0.869	0.0007/<0.0001/0.0002

Appendix Table S4

Figure	Analysis of normality	Statistical method	Power	p value
Fig 3 D	Yes	two-tailed unpaired Student's t test	0.051/0.365/0.052/0.141/0.948/0.052/0.055/0.883/0.097/0.058/0.999	0.9218/0.1434/0.8831/0.3615/0.0036/0.8881/0.8144/0.0336/0.4922/0.7670/0.0096
Fig 3 E	Yes	two-tailed unpaired Student's t test	0.115	0.5249
Fig 3 G	Yes	two-tailed unpaired Student's t test	1.000/1.000	0.0001/<0.0001
Fig 3 H	Yes	two-tailed unpaired Student's t test	0.329	0.1494

Appendix Table S5

Figure	Analysis of normality	Statistical method	Power	p value
Fig 4 G	Yes	two-way ANOVA followed by the Tukey test	0.892	<0.0001

Appendix Table S6

Figure	Analysis of normality	Statistical method	Power	p value
Fig 6 E	Yes	two-way ANOVA followed by the Tukey test	0.858/0.801/0.847	0.0160/0.9883/0.0236
Fig 6 F	Yes	two-way ANOVA followed by the Tukey test	0.907/0.803/0.863	0.0075/0.8458/0.0226
Fig 6 G	Yes	two-way ANOVA followed by the Tukey test	0.922/0.810/0.823	0.0085/0.8739/0.0229

Appendix Table S7

Figure	Analysis of normality	Statistical method	Power	p value
Fig 7 B	Yes	two-tailed unpaired Student's t test	1.000	<0.0001
Fig 7 D	Yes	two-way ANOVA followed by the Tukey test	0.943/0.882	0.0040/0.0310
Fig 7 E	Yes	two-way ANOVA followed by the Tukey test	1.000/0.802	0.0002/0.0418
Fig 7 F	Yes	two-way ANOVA followed by the Tukey test	1.000/0.997	<0.0001/0.0002
Fig 7 G (*Il1b*/*Il6*/*Tnf*)	Yes	two-way ANOVA followed by the Tukey test	0.730/0.995/1.000	0.0058/<0.0001/<0.0001
Fig 7 I	Yes	two-way ANOVA followed by the Tukey test	0.875	0.0006
Fig 7 L	Yes	two-way ANOVA followed by the Tukey test	0.915	0.0114

Appendix Table S8

Figure	Analysis of normality	Statistical method	Power	p value
Fig 8 B	Yes	two-way ANOVA followed by the Tukey test	0.801	<0.0001
Fig 8 C	Yes	two-way ANOVA followed by the Tukey test	0.803	0.0001
Fig 8 F	Yes	two-tailed unpaired Student's t test	0.853	0.0094
Fig 8 G	Yes	two-tailed unpaired Student's t test	1.000	0.0006
Fig 8 H	Yes	two-way ANOVA followed by the Tukey test	0.974/1.000/0.868/ 0.906/0.821/1.000	<0.0001/<0.0001/0.0018/ 0.0087/0.0459/<0.0001

Appendix Table S9

Figure	Analysis of normality	Statistical method	Power	p value
Fig 9 D	Yes	two-tailed unpaired Student's t test	0.800/1.000/1.000	0.8653/0.0038/0.0053
Fig 9 G	Yes	two-tailed unpaired Student's t test	0.901	0.0245
Fig 9 J	Yes	two-tailed unpaired Student's t test	0.928/1.000/1.000	0.0674/<0.0001/0.0014
Fig 9 L	Yes	two-tailed unpaired Student's t test	1.000	0.0347

Appendix Table S10

Figure	Analysis of normality	Statistical method	Power	p value
Appendix Fig S1 B	No	Mann-Whitney U test	0.800	0.0547

Appendix Table S11

Figure	Analysis of normality	Statistical method	Power	p value
Appendix Fig S2 C	Yes	two-tailed unpaired Student's t test	1.000	0.0043
Appendix Fig S2 E	Yes	two-tailed unpaired Student's t test	1.000	0.0057
Appendix Fig S2 H	Yes	two-tailed unpaired Student's t test	0.800	0.9037

Appendix Table S12

Figure	Analysis of normality	Statistical method	Power	p value
Appendix Fig S3 B	Yes	two-way ANOVA followed by the Tukey test	1.000	0.0273

Appendix Table S13

Figure	Analysis of normality	Statistical method	Power	p value
Appendix Fig S6 C	Yes	two-tailed unpaired Student's t test	1.000/1.000/0.800/0.842/0.934/0.800	0.0315/0.0014/0.4760/0.0773/0.0901/0.9138
Appendix Fig S6 E	Yes	two-tailed unpaired Student's t test	1.000/1.000/0.802/0.886	0.0003/0.0001/0.8443/0.0703
Appendix Fig S6 I	Yes	two-tailed unpaired Student's t test	1.000/1.000	0.0164/0.0214
