# Supplementary material for: OTUB1 non-canonically inhibits TAB2 ubiquitination to govern microglia-mediated neuroinflammation
Source: EMBO Mol Med. 2026 Jul 2;18(8):3137–56. doi: 10.1038/s44321-026-00473-x (PMC13470327; doi:10.1038/s44321-026-00473-x)
Supplement: Supplementary file 2 [file 44321_2026_473_MOESM2_ESM.pdf]

EMBO Molecular Medicine

# OTUB1 non-canonically inhibits TAB2 ubiquitination to govern microglia-mediated neuroinflammation

Zijun Cao, Zhenhu Zhu, Ping Zeng, Fuqi Mei, Deqi Wang, Jun Xu, Yanqi Xu, Kangmin Chen, Chushan Wei, Jiangyun Shen, Keshuo Jin, Jiaqing Chen, Zhongding Li, Baohua Liu, Dirk Schlüter, Jingyong Huang, and Xu Wang

*Corresponding author: Xu Wang (wangxu@ojlab.ac.cn)*



## Review Timeline:

| | |
|---|---|
| Submission Date: | 1st Dec 25 |
| Editorial Decision: | 9th Jan 26 |
| Revision Received: | 9th May 26 |
| Editorial Decision: | 3rd Jun 26 |
| Revision Received: | 4th Jun 26 |
| Accepted: | 9th Jun 26 |

*Editor: Jingyi Hou*

## Transaction Report:

(Note: With the exception of the correction of typographical or spelling errors that could be a source of ambiguity, letters and reports are not edited. Depending on transfer agreements, referee reports obtained elsewhere may or may not be included in this compilation. Referee reports are anonymous unless the Referee chooses to sign their reports.)

9th Jan 2026

Dear Prof. Wang,

Thank you for submitting your work to EMBO Molecular Medicine. We have now received reports from the two referees who evaluated your manuscript. As outlined below, while Referee #2 is generally supportive of publication, Referee #1 raises several concerns regarding conceptual novelty, potential overstatements, several aspects of the experimental design, and the preliminary nature of the translational advance.

During our pre-decision cross-commenting process (in which the referees are given an opportunity to provide additional feedback, including comments on each other's reports), both referees agreed that the authors should be given the opportunity to revise the manuscript, as the major concerns are in principle addressable. However, Referee #1 reiterated that "the manuscript needs to be substantially revised by adding new data and toning down several of the main claims," and emphasized that the revised manuscript should be critically re-reviewed to ensure that the key concerns have been adequately addressed.

In light of these assessments, we invite you to submit a major revision of your manuscript. Please ensure that all issues raised by the referees are carefully addressed.

As you may already know, our editorial policy allows in principle a single round of major revision, so it is essential to provide responses to the referees' comments that are as complete as possible. Please feel free to contact me in case you would like to discuss in further detail any of the issues raised by the referees.

EMBO Molecular Medicine has a "scooping protection" policy, whereby similar findings that are published by others during review or revision are not a criterion for rejection. Should you decide to submit a revised version, I do ask that you get in touch after three months if you have not completed it, to update us on the status.

Please also contact us as soon as possible if similar work is published elsewhere. If other work is published, we may not be able to extend the revision period beyond three months.

Please read below for important editorial formatting and consult our author's guidelines for proper formatting of your revised article for EMBO Molecular Medicine.

I look forward to receiving your revised manuscript.

Kind regards,
Jingyi

Jingyi Hou
Senior Editor
EMBO Molecular Medicine

*****
When preparing your revised manuscript, please refer to our guidelines: https://link.springer.com/journal/44321/submission-guidelines#cms-Revised-submissions. We perform an initial quality control of all revised manuscripts before re-review; failure to include requested items will delay the evaluation of your revision.

We require:

1) A .docx formatted version of the manuscript text (including legends for main figures, EV figures and tables). Please make sure that the changes are highlighted to be clearly visible.

2) Individual production quality figure files as .eps, .tif, .jpg (one file per figure). For guidance, download the 'Figure Guide PDF': https://media.springernature.com/original/springer-cms/rest/v1/content/27825798/data/v1.

3) A .docx formatted letter INCLUDING the reviewers' reports and your detailed point-by-point responses to their comments. As part of the EMBO Press transparent editorial process, the point-by-point response is part of the Review Process File (RPF), which will be published alongside your paper.

4) A complete author checklist, which you can download from our author guidelines. Please insert information in the checklist that is also reflected in the manuscript. The completed author checklist will also be part of the RPF.

5) Please note that all corresponding authors are required to supply an ORCID ID for their name upon submission of a revised manuscript.

6) It is mandatory to include a 'Data Availability' section after the Materials and Methods. Before submitting your revision, primary datasets produced in this study need to be deposited in an appropriate public database, and the accession numbers and database listed under 'Data Availability'. Please remember to provide a reviewer password if the datasets are not yet public.

In case you have no data that requires deposition in a public database, please state so in this section. Note that the Data Availability Section is restricted to new primary data that are part of this study.

7) For data quantification: please specify the name of the statistical test used to generate error bars and P values, the number (n) of independent experiments (specify technical or biological replicates) underlying each data point and the test used to calculate p-values in each figure legend. The figure legends should contain a basic description of n, P and the test applied. Graphs must include a description of the bars and the error bars (s.d., s.e.m.).

8) At EMBO Press we ask authors to provide source data for the main manuscript figures. You will receive a separate email with instructions for providing source data with your revised manuscript, including how to upload and organize the files.

9) Our journal encourages inclusion of *data citations in the reference list* to directly cite datasets that were re-used and obtained from public databases. Data citations in the article text are distinct from normal bibliographical citations and should directly link to the database records from which the data can be accessed. In the main text, data citations are formatted as follows: "Data ref: Smith et al, 2001" or "Data ref: NCBI Sequence Read Archive PRJNA342805, 2017". In the Reference list, data citations must be labeled with "[DATASET]". A data reference must provide the database name, accession number/identifiers and a resolvable link to the landing page from which the data can be accessed at the end of the reference.

10) We replaced Supplementary Information with Expanded View (EV) Figures and Tables that are collapsible/expandable online. A maximum of 5 EV Figures can be typeset. EV Figures should be cited as 'Figure EV1, Figure EV2" etc... in the text and their respective legends should be included in the main text after the legends of regular figures.

- For the figures that you do NOT wish to display as Expanded View figures, they should be bundled together with their legends in a single PDF file called *Appendix*, which should start with a short Table of Content. Appendix figures should be referred to in the main text as: "Appendix Figure S1, Appendix Figure S2" etc.

- Additional Tables/Datasets should be labeled and referred to as Table EV1, Dataset EV1, etc. Legends have to be provided in a separate tab in case of .xls files. Alternatively, the legend can be supplied as a separate text file (README) and zipped together with the Table/Dataset file.

11) The paper explained: EMBO Molecular Medicine articles are accompanied by a summary of the articles to emphasize the major findings in the paper and their medical implications for the non-specialist reader. Please provide a draft summary of your article highlighting

- the medical issue you are addressing,

- the results obtained and

- their clinical impact.

This may be edited to ensure that readers understand the significance and context of the research. Please refer to any of our published articles for an example.

12) Author contributions: You will be asked to provide CRediT (Contributor Role Taxonomy) terms in the submission system. These replace a narrative author contribution section in the manuscript.

13) A Conflict of Interest statement should be provided in the main text.

14) Please provide a 'Synopsis' to further enhance discoverability. Synopses are displayed on the journal webpage and are freely accessible to all readers. They include a short stand first (maximum of 300 characters, including space) as well as 2-5 one-sentences bullet points that summarizes the paper. Please write the bullet points to summarize the key NEW findings. They should be designed to be complementary to the abstract - i.e. not repeat the same text. We encourage inclusion of key

acronyms and quantitative information (maximum of 30 words / bullet point). Please use the passive voice. Please attach these in a separate file or send them by email, we will incorporate them accordingly.

Please also suggest a visual abstract to illustrate your article as a PNG file 550 px wide x 300-600 px high.

15) Please download and fill our Reagents and Tools Table template (.docx), which you can find in our author guidelines: https://link.springer.com/journal/44320/submission-guidelines#structuredmethods.

When submitting your revised manuscript, please do not include the Reagents and Tools Table in the Methods section of the manuscript but upload it as a separate file choosing the file type "Reagent Table".

***** Reviewer's comments *****

Referee #1 (Remarks for Author):

This manuscript investigates the role of the deubiquitinase OTUB1 in regulating microglia-mediated neuroinflammation, proposing a non-canonical mechanism by which OTUB1 stabilizes TAB2 and thereby amplifies TLR-NF-κB/MAPK signaling. Using a combination of microglia-specific genetic deletion, in vitro mechanistic studies, and several disease models (ischemic stroke, LPS-induced sickness/depression-like behavior, and EAE), the authors present a coherent dataset suggesting that OTUB1 promotes pro-inflammatory microglial activation. several key claims extend beyond what is directly demonstrated by the data, and important aspects of experimental design, specificity, and translational relevance remain insufficiently controlled or discussed. While the core finding that OTUB1 modulates TAB2 stability in microglia is interesting, the overall conceptual novelty is rather incremental, and the therapeutic conclusions are currently largely overstated.

Major Comments
1. The identification of OTUB1 as a positive regulator of TLR signaling via stabilization of UBC13 and TAB2 in microglia extends prior work on OTUB1 in dendritic cells and astrocytes. While the demonstration of TAB2 as a non-canonical OTUB1 target is mechanistically solid, the broader conceptual advance is limited. Multiple studies have already established TAB2 as a ubiquitination-regulated signaling hub in TLR pathways, and several DUBs have been shown to converge on the same signaling axis in microglia. The manuscript would benefit from a more restrained framing that emphasizes refinement of existing signaling paradigms rather than positioning OTUB1 as a fundamentally new "central" regulator of neuroinflammation.

2. Experimental design : The use of Cx3cr1-CreERT2 to delete OTUB1 in microglia is appropriate but raises unresolved concerns regarding specificity. Given the known recombination in peripheral myeloid populations and border-associated macrophages, the conclusion that the observed phenotypes are strictly microglia-intrinsic is not fully supported. No fate-mapping or peripheral immune profiling is provided to exclude contributions from monocytes or macrophages, particularly in the stroke and EAE models where peripheral immune infiltration is substantial. Additional controls or a more cautious interpretation are required.

3. The reduction in infarct size, behavioral deficits, and inflammatory markers in Otub1CKO mice is convincing at a descriptive level. However, the causal link between the proposed OTUB1-TAB2 mechanism and the in vivo protection is not directly demonstrated. No rescue experiments (e.g., TAB2 re-expression or stabilization in OTUB1-deficient microglia) are provided, leaving open the possibility that parallel OTUB1-dependent pathways contribute to the phenotype. As a result, the mechanistic conclusions drawn from the in vitro data are extrapolated too strongly to the in vivo disease models.

4. Use of BV2 cells - While the authors include primary microglia for cytokine readouts, most mechanistic experiments rely heavily on BV2 cells. Given the known limitations of BV2 cells in faithfully recapitulating primary microglial signaling and ubiquitin dynamics, the strength of the mechanistic conclusions would be improved by additional validation in primary microglia, particularly for TAB2 ubiquitination and degradation assays.

5. The interpretation of LPS-induced "depression-like behavior" is overstated. Acute systemic LPS administration induces sickness behavior that overlaps with, but is not equivalent to, depressive phenotypes. The manuscript does not adequately disentangle sickness effects from affective behavior, nor does it include controls for locomotor impairment. Claims regarding depression-like behavior should therefore be substantially toned down.

6. The therapeutic conclusions are the weakest aspect of the study. OTUB1/USP8-IN-1 is a dual inhibitor, and despite the authors' efforts to address USP8 involvement, the data do not fully exclude off-target or combinatorial effects in vivo. Moreover, no pharmacokinetic, brain exposure, or target engagement data are provided. The assertion that OTUB1 represents a promising therapeutic target for neuroinflammatory diseases is therefore premature and should be reframed as a proof-of-principle observation rather than a translational advance.
7. Statistics: Justification of power calculations is lacking, particularly for behavioral experiments. The statistical approach relies predominantly on parametric tests without clear evidence that assumptions are met.

Minor Comments
The manuscript frequently uses broad terms such as "universal regulator" and "essential mediator," which are not justified by the data and should be replaced by more precise language. Several figures would benefit from clearer indication of biological versus technical replicates. Finally, the Discussion would benefit from a more explicit acknowledgment of the redundancy and compensatory mechanisms within ubiquitin-dependent TLR signaling.

Referee #2 (Remarks for Author):

Microglia contribute to neuroinflammation under pathological conditions, thereby driving the pathogenesis and progression of various central nervous system (CNS) diseases. Cao et al. identified the deubiquitinating enzyme OTUB1 as a pivotal regulator of microglial activation and neuroinflammation. Microglia-specific deletion of OTUB1 in mice significantly ameliorated ischemic brain injury by attenuating pro-inflammatory activation. Mechanistically, OTUB1 enhances Toll-like receptor (TLR) signaling by stabilizing UBC13 and TAB2, thereby amplifying cytokine induction. Notably, OTUB1 inhibits the proteasomal degradation of TAB2 by suppressing its K48-linked ubiquitination in a catalytic activity-independent manner. Furthermore, microglia-restricted OTUB1 deficiency alleviated lipopolysaccharide-induced depression-like behavior and experimental autoimmune encephalomyelitis in mice by reducing neuroinflammation. Pharmacological inhibition of OTUB1 also significantly mitigated ischemic stroke injury. This study provides important insights into the regulation of microglial function in neuroinflammation, with potential therapeutic implications. The following points may help further strengthen the work:
1. To confirm the deletion of OTUB1 in microglia, the authors need to perform qPCR analysis on sorted CNS microglia and western blot analysis of cultured primary microglia from WT and OTUB1-cKO mice.
2. The authors showed reduction of Il1b, Il6, and Tnf mRNA levels in Otub1-deficient BV2 cells and primary microglia (Figure 2H and I). The author needs to confirm the protein levels of these cytokines using ELISA.
3. In the EAE model, the authors claim that OTUB1 deletion decreased lymphocyte infiltration in the CNS (Figure 7D and E). The authors should demonstrate the infiltrating T cells, including Th1 and Th17, using flow cytometry.
4. The authors need to add a quantification and statistical analysis of Figure 6A and J.
5. The exact dosage of the OTUB1/USP8 inhibitor used for in vivo experiments should be clearly stated. Additionally, the conclusion that "pharmacological inhibition of OTUB1 confers protection against ischemic brain injury by downregulating microglial activation" requires caution, as the inhibitor may act on various cell types in vivo, not exclusively on microglia.

***** Reviewer's comments *****

Referee #1 (Remarks for Author):

This manuscript investigates the role of the deubiquitinase OTUB1 in regulating microglia-mediated neuroinflammation, proposing a non-canonical mechanism by which OTUB1 stabilizes TAB2 and thereby amplifies TLR-NF-κB/MAPK signaling. Using a combination of microglia-specific genetic deletion, in vitro mechanistic studies, and several disease models (ischemic stroke, LPS-induced sickness/depression-like behavior, and EAE), the authors present a coherent dataset suggesting that OTUB1 promotes pro-inflammatory microglial activation. several key claims extend beyond what is directly demonstrated by the data, and important aspects of experimental design, specificity, and translational relevance remain insufficiently controlled or discussed. While the core finding that OTUB1 modulates TAB2 stability in microglia is interesting, the overall conceptual novelty is rather incremental, and the therapeutic conclusions are currently largely overstated.

Major Comments

1. The identification of OTUB1 as a positive regulator of TLR signaling via stabilization of UBC13 and TAB2 in microglia extends prior work on OTUB1 in dendritic cells and astrocytes. While the demonstration of TAB2 as a non-canonical OTUB1 target is mechanistically solid, the broader conceptual advance is limited. Multiple studies have already established TAB2 as a ubiquitination-regulated signaling hub in TLR pathways, and several DUBs have been shown to converge on the same signaling axis in microglia. The manuscript would benefit from a more restrained framing that emphasizes refinement of existing signaling paradigms rather than positioning OTUB1 as a fundamentally new "central" regulator of neuroinflammation.

**Response:** TAB2 is a key signaling hub upstream of the pro-inflammatory NF-κB and MAPK signaling pathways. As mentioned by the reviewer and stated by us in the Discussion, several studies have shown that ubiquitination regulates both the activity and stability of TAB2. Notably, pivotal signaling molecules are usually regulated by multiple DUBs to ensure their optimal function. In the present study, we identified OTUB1 as a new DUB modulating TAB2 stability. Importantly, in addition to TAB2, UBC13 was also regulated by OTUB1 in TLR signaling. Therefore, OTUB1 potentiates TLR signaling by increasing the expression of two key signaling molecules. This “double-safety” mechanism positions OTUB1 as an important regulator of

TLR-mediated inflammatory responses. We agree with the reviewer that it is more appropriate to tone down the expression regarding the role of OTUB1 in neuroinflammation; therefore, we have modified the manuscript according to the reviewer's suggestions listed below.

2. Experimental design : The use of Cx3cr1-CreERT2 to delete OTUB1 in microglia is appropriate but raises unresolved concerns regarding specificity. Given the known recombination in peripheral myeloid populations and border-associated macrophages, the conclusion that the observed phenotypes are strictly microglia-intrinsic is not fully supported. No fate-mapping or peripheral immune profiling is provided to exclude contributions from monocytes or macrophages, particularly in the stroke and EAE models where peripheral immune infiltration is substantial. Additional controls or a more cautious interpretation are required.

**Response:** We agree with the reviewer that Cx3cr1 is expressed not only in microglia but also in macrophages. To circumvent this problem, we used the tamoxifen-induced Cx3cr1-CreERT2 system to specifically, at least relatively, delete OTUB1 in microglia. Although this system is not perfect, it is the best technique that we could apply to study the microglia-specific function of OTUB1 *in vivo*. We conducted Western blot and qRT-PCR to analyze OTUB1 expression in peritoneal macrophages and bone marrow-derived macrophages of *Otub1*$^{flox}$ and *Otub1*$^{CKO}$ mice after tamoxifen injection. The results show that OTUB1 expression in peripheral macrophages was comparable between *Otub1*$^{flox}$ and *Otub1*$^{CKO}$ mice (new Appendix Fig. S2F-H). In addition, we sorted microglia from tamoxifen-treated *Otub1*$^{flox}$ and *Otub1*$^{CKO}$ mice and found that *Otub1* mRNA levels were significantly reduced in microglia of *Otub1*$^{CKO}$ mice (new Appendix Fig. S2C). Moreover, after 4-hydroxytamoxifen treatment, cultured primary microglia from *Otub1*$^{CKO}$ mice showed significantly reduced OTUB1 expression compared with those from *Otub1*$^{flox}$ mice (new Appendix Fig. S2D-E). Therefore, the observed phenotypes in this study should be largely attributed to OTUB1 deficiency in microglia.

3. The reduction in infarct size, behavioral deficits, and inflammatory markers in Otub1CKO mice is convincing at a descriptive level. However, the causal link between the proposed OTUB1-TAB2 mechanism and the in vivo protection is not directly demonstrated. No rescue experiments (e.g., TAB2 re-expression or stabilization in OTUB1-deficient microglia) are provided, leaving open the possibility that parallel OTUB1-dependent pathways contribute to the phenotype. As a result, the mechanistic conclusions drawn from the in vitro data are extrapolated too strongly to the in vivo disease models.

**Response:** We thank the reviewer for this constructive suggestion. As suggested, we ectopically

expressed FLAG-tagged TAB2 in microglia of *Otub1*$^{flox}$ and *Otub1*$^{CKO}$ mice through stereotactic injection of adeno-associated virus serotype 11 carrying Iba-1 promoter and FLAG-TAB2 sequences. Expression of FLAG-TAB2 in OTUB1-deficient microglia significantly increased cerebral infarct size and behavioral impairment after MCAO (new Fig. 6A-G). Moreover, ectopic expression of TAB2 blunted the differences in infarct volume and behavioral deficits between *Otub1*$^{flox}$ and *Otub1*$^{CKO}$ mice (new Fig. 6D-G), verifying that OTUB1 affects stroke phenotypes by regulating TAB2.

4. Use of BV2 cells - While the authors include primary microglia for cytokine readouts, most mechanistic experiments rely heavily on BV2 cells. Given the known limitations of BV2 cells in faithfully recapitulating primary microglial signaling and ubiquitin dynamics, the strength of the mechanistic conclusions would be improved by additional validation in primary microglia, particularly for TAB2 ubiquitination and degradation assays.

**Response:** We agree with the reviewer that key mechanistic results obtained with BV2 cells should be validated in primary microglia. As requested by the reviewer, we analyzed the ubiquitination and degradation of TAB2 in primary microglia. In good agreement with results obtained using BV2 cells, the new results show that, in primary microglia, OTUB1 stabilized TAB2 by inhibiting its proteasomal degradation (new Fig. 4L-M). In addition, OTUB1 deficiency markedly increased the K48 ubiquitination of TAB2 in primary microglia (new Fig. 5D).

5. The interpretation of LPS-induced "depression-like behavior" is overstated. Acute systemic LPS administration induces sickness behavior that overlaps with, but is not equivalent to, depressive phenotypes. The manuscript does not adequately disentangle sickness effects from affective behavior, nor does it include controls for locomotor impairment. Claims regarding depression-like behavior should therefore be substantially toned down.

**Response:** We thank the reviewer for this important suggestion. To be more precise, we have changed “depression-like behavior” to “sickness behavior”, lines 36, 102, 264, 271, 293, 495, 911-912.

6. The therapeutic conclusions are the weakest aspect of the study. OTUB1/USP8-IN-1 is a dual inhibitor, and despite the authors' efforts to address USP8 involvement, the data do not fully exclude off-target or combinatorial effects in vivo. Moreover, no

pharmacokinetic, brain exposure, or target engagement data are provided. The assertion that OTUB1 represents a promising therapeutic target for neuroinflammatory diseases is therefore premature and should be reframed as a proof-of-principle observation rather than a translational advance.

**Response:** The reviewer is correct that we cannot fully exclude other effects of OTUB1/USP8-IN-1 since it is not a specific OTUB1 inhibitor. We honestly admitted this shortcoming and discussed this problem in the Discussion, pointing out that this is a limitation of our study, lines 395-399. As suggested by the reviewer, we analyzed the pharmacokinetics, brain exposure, and target engagement of OTUB1/USP8-IN-1. As shown in the new Appendix Fig. S6G, OTUB1/USP8-IN-1 rapidly entered the brain at 1 and 3 hours after intraperitoneal administration. In addition, OTUB1/USP8-IN-1 treatment significantly reduced protein levels of UBC13, TAB2, and EGFR in the brain (new Fig. 9I-J, new Appendix Fig. S6H-I), indicating that it effectively inhibits OTUB1 and USP8 in the brain. Moreover, instead of stating that "OTUB1 as a therapeutic target for treating TLR-associated neuroinflammatory diseases", we have rephrased the sentence as "These findings reveal an important role of OTUB1 in potentiating microglial activation and neuroinflammation, providing a proof-of-principle observation for targeting OTUB1 in the treatment of TLR-associated neuroinflammatory diseases", lines 38-41.

7. Statistics: Justification of power calculations is lacking, particularly for behavioral experiments. The statistical approach relies predominantly on parametric tests without clear evidence that assumptions are met.

**Response:** To improve the transpanrency of statistics, we have added statistical tables for each panel in each figure, specifying analysis of normality, statistical methods, power, and exact p values (new Appendix Table S2-S13).

Minor Comments

The manuscript frequently uses broad terms such as "universal regulator" and "essential mediator," which are not justified by the data and should be replaced by more precise language. Several figures would benefit from clearer indication of biological versus technical replicates. Finally, the Discussion would benefit from a more explicit acknowledgment of the redundancy and compensatory mechanisms within ubiquitin-dependent TLR signaling.

**Response:** As suggested by the reviewer, we have toned down the expression and removed "universal regulator" and "essential mediator" to make the statements more precise, lines 109,

294. In addition, we have clearly indicated biological versus technical replicates for each figure in Materials and methods and Figure legends. Moreover, as suggested by the reviewer, we have explicitly acknowledged the complex regulation of TAB2 by ubiquitination, lines 365-368.

Referee #2 (Remarks for Author):

Microglia contribute to neuroinflammation under pathological conditions, thereby driving the pathogenesis and progression of various central nervous system (CNS) diseases. Cao et al. identified the deubiquitinating enzyme OTUB1 as a pivotal regulator of microglial activation and neuroinflammation. Microglia-specific deletion of OTUB1 in mice significantly ameliorated ischemic brain injury by attenuating pro-inflammatory activation. Mechanistically, OTUB1 enhances Toll-like receptor (TLR) signaling by stabilizing UBC13 and TAB2, thereby amplifying cytokine induction. Notably, OTUB1 inhibits the proteasomal degradation of TAB2 by suppressing its K48-linked ubiquitination in a catalytic activity-independent manner. Furthermore, microglia-restricted OTUB1 deficiency alleviated lipopolysaccharide-induced depression-like behavior and experimental autoimmune encephalomyelitis in mice by reducing neuroinflammation. Pharmacological inhibition of OTUB1 also significantly mitigated ischemic stroke injury. This study provides important insights into the regulation of microglial function in neuroinflammation, with potential therapeutic implications. The following points may help further strengthen the work:

1. To confirm the deletion of OTUB1 in microglia, the authors need to perform qPCR analysis on sorted CNS microglia and western blot analysis of cultured primary microglia from WT and OTUB1-cKO mice.

**Response:** As suggested by the reviewer, we sorted microglia from tamoxifen-treated *Otub1*$^{flox}$ and *Otub1*$^{CKO}$ mice and analyzed *Otub1* mRNA expression by qRT-PCR. The results show that *Otub1* mRNA levels were significantly reduced in microglia of tamoxifen-treated *Otub1*$^{CKO}$ mice (new Appendix Fig. S2C). In addition, we analyzed OTUB1 expression in cultured primary microglia isolated from *Otub1*$^{flox}$ and *Otub1*$^{CKO}$ mice by Western blot. After 4-hydroxytamoxifen treatment, primary microglia from *Otub1*$^{CKO}$ mice showed significantly reduced OTUB1 expression compared with those from *Otub1*$^{flox}$ mice (new Appendix Fig. S2D-E). These new results are consistent with the previous immunofluorescence results (Appendix Fig. S2B), confirming the deletion of OTUB1 in microglia of tamoxifen-treated *Otub1*$^{CKO}$ mice.

2. The authors showed reduction of Il1b, Il6, and Tnf mRNA levels in Otub1-deficient BV2 cells and primary microglia (Figure 2H and I). The author needs to confirm the protein levels of these cytokines using ELISA.

**Response:** As requested by the reviewer, we analyzed these cytokines by ELISA. In agreement with the qRT-PCR results, OTUB1 deletion significantly reduced protein levels of IL-1β, IL-6, and TNF-α in the culture medium of BV2 cells and primary microglia after LPS stimulation (new Fig. 2I, 2K).

3. In the EAE model, the authors claim that OTUB1 deletion decreased lymphocyte infiltration in the CNS (Figure 7D and E). The authors should demonstrate the infiltrating T cells, including Th1 and Th17, using flow cytometry.

**Response:** We agree with the reviewer that Th1 and Th17 are critical effector cell populations in EAE. Therefore, we analyzed infiltrating Th1 and Th17 cells in the spinal cord using flow cytometry. Compared with *Otub1*$^{flox}$ EAE mice, *Otub1*$^{CKO}$ EAE mice had significantly reduced numbers of Th1 and Th17 cells in the spinal cord (new Fig. 8F-G).

4. The authors need to add a quantification and statistical analysis of Figure 6A and J.

**Response:** As requested by the reviewer, we have added statistical analysis for Fig. 6A and 6J. The new quantitative data are displayed as the new Fig. 7B and 7L.

5. The exact dosage of the OTUB1/USP8 inhibitor used for in vivo experiments should be clearly stated. Additionally, the conclusion that "pharmacological inhibition of OTUB1 confers protection against ischemic brain injury by downregulating microglial activation" requires caution, as the inhibitor may act on various cell types in vivo, not exclusively on microglia.

**Response:** We thank the reviewer for these suggestions. The exact dosage of OTUB1/USP8-IN-1 used for *in vivo* experiments was clearly stated in the new Fig. 9F. In addition, we have rephrased the statement as “pharmacological inhibition of OTUB1 confers protection against ischemic brain injury, which should be largely attributed to the downregulation of microglial activation”, lines 324-325.

3rd Jun 2026

Dear Prof. Wang,

Thank you for the submission of your revised manuscript to EMBO Molecular Medicine. We have now received the enclosed reports from the two reviewers who were asked to reassess your manuscript. As you will see, both referees agree that the revision has improved the study, although Reviewer #1 still raises several concerns regarding overstatement and requests more cautious wording.

Before I can accept your manuscript, pending the amendments outlined below, please address the remaining comment from Reviewer #1. Please use track changes, as we need to review the modifications.

On a more editorial level:

1. Please provide an institutional email address for the corresponding author.

2. Please provide exact p values in the legends of figures 1B, E, G; 2B, H, I; 3G, 4G, 7E, G; 8H, 9J.

3. Please move "The paper explained"to the main manuscript file.

4. We have updated the dimension requirements for the visual abstract. Please provide it as a PNG file with exact dimensions of 550 px (width) × 300 px (height).

5. Source Data formatting

- Source data for main figures: should be uploaded as one (zipped) file /figure, and named as "manuscriptID_SourceDataForFigure x"
- Source data for supplementary figures can be combined in one file.

6. During our routine image check, we noticed potential figure reuse between Figure 1Q and Figure 9N. Please review these panels. If the figure or sample has been reused, this should be explicitly stated in the figure legend.

7. "Materials and methods" should be renamed to "Methods".

8. The manuscript sections should be in the following order: Title page - Abstract - Introduction - Results - Discussion - Methods -Data Availability - Acknowledgments - Disclosure Statement & Competing Interests - References - Figure Legends - (Main Tables with legends if applicable) - Expanded View Figure Legends.

I look forward to reading a new revised version of your manuscript as soon as possible.

Kind regards,
Jingyi

Jingyi Hou
Senior Editor
EMBO Molecular Medicine

*** Instructions to submit your revised manuscript ***

*** PLEASE NOTE *** As part of the EMBO Publications transparent editorial process initiative (see our Editorial at https://www.embopress.org/doi/pdf/10.1002/emmm.201000094), EMBO Molecular Medicine will publish online a Review Process File to accompany accepted manuscripts.

In the event of acceptance, this file will be published in conjunction with your paper and will include the anonymous referee reports, your point-by-point response and all pertinent correspondence relating to the manuscript. If you do NOT want this file to be published, please inform the editorial office at contact@embomolmed.org.

*****

***** Reviewer's comments *****

Referee #1 (Remarks for Author):

The revised manuscript has been substantially strengthened and addresses several of the major concerns raised in the initial review. In particular, the newly added TAB2 rescue experiments in vivo significantly improve the mechanistic support for the proposed OTUB1-TAB2 signaling axis in ischemic injury, and the additional validation of key ubiquitination and degradation findings in primary microglia increases confidence in the biological relevance of the mechanistic conclusions. The authors also appropriately corrected the interpretation of the acute LPS model from "depression-like behavior" to "sickness behavior," improved statistical transparency, and moderated several overstated conclusions. Overall, the revision represents a serious and scientifically meaningful effort that considerably improves the manuscript.

That said, some issues remain only partially resolved:
First, although the additional analyses of peripheral macrophages and sorted microglia improve confidence in the specificity of the Cx3cr1-CreERT2 model, the interpretation remains somewhat broader than fully supported by the data. The possibility of contributions from border-associated macrophages or transient peripheral myeloid recombination cannot be entirely excluded, particularly in the stroke and EAE settings. The manuscript should therefore maintain cautious wording regarding strict microglia specificity.
Second, despite improved framing, the therapeutic conclusions remain somewhat overstated. The additional BBB penetration and target engagement data are useful; however, OTUB1/USP8-IN-1 remains a dual inhibitor, and the manuscript still occasionally attributes the observed effects too directly and specifically to OTUB1 inhibition. In particular, statements such as "OTUB1 may become a new and efficacious therapeutic target" remain stronger than justified by the current proof-of-principle data and should be moderated further.
Third, the novelty framing remains slightly inflated in some sections of the manuscript. The identification of TAB2 as an OTUB1-regulated substrate in microglia is biologically interesting and mechanistically relevant, but the overall conceptual advance remains an extension and refinement of existing ubiquitin-dependent TLR signaling paradigms rather than a fundamentally new signaling principle. Terms such as "pivotal regulator" should therefore be used with caution.

Referee #2 (Remarks for Author):

The authors have sufficiently addressed my concerns. I suggested to accept this manuscript.

***** Reviewer's comments *****

Referee #1 (Remarks for Author):

The revised manuscript has been substantially strengthened and addresses several of the major concerns raised in the initial review. In particular, the newly added TAB2 rescue experiments in vivo significantly improve the mechanistic support for the proposed OTUB1-TAB2 signaling axis in ischemic injury, and the additional validation of key ubiquitination and degradation findings in primary microglia increases confidence in the biological relevance of the mechanistic conclusions. The authors also appropriately corrected the interpretation of the acute LPS model from "depression-like behavior" to "sickness behavior," improved statistical transparency, and moderated several overstated conclusions. Overall, the revision represents a serious and scientifically meaningful effort that considerably improves the manuscript.

That said, some issues remain only partially resolved:
First, although the additional analyses of peripheral macrophages and sorted microglia improve confidence in the specificity of the Cx3cr1-CreERT2 model, the interpretation remains somewhat broader than fully supported by the data. The possibility of contributions from border-associated macrophages or transient peripheral myeloid recombination cannot be entirely excluded, particularly in the stroke and EAE settings. The manuscript should therefore maintain cautious wording regarding strict microglia specificity.

**Response:** As suggested by the reviewer, we mentioned the potential contribution of OTUB1 in peripheral myeloid cells to neuroinflammation by adding "Although OTUB1 was mainly deleted in microglia in Cx3cr1-Cre$^{ERT2}$ *Otub1*$^{fl/fl}$ mice after tamoxifen treatment, perturbed OTUB1 in peripheral myeloid populations and border-associated macrophages may also affect neuroinflammation" to the Discussion, line 330-333.

Second, despite improved framing, the therapeutic conclusions remain somewhat overstated. The additional BBB penetration and target engagement data are useful; however, OTUB1/USP8-IN-1 remains a dual inhibitor, and the manuscript still occasionally attributes the observed effects too directly and specifically to OTUB1 inhibition. In particular, statements such as "OTUB1 may become a new and efficacious therapeutic target" remain stronger than justified by the current proof-of-principle data and should be moderated further.

**Response:** As suggested by the reviewer, we have changed the sentence to "The development of specific inhibitors for OTUB1 will be beneficial for the further validation of OTUB1 as a therapeutic target for neuroinflammatory diseases", lines 414-416.

Third, the novelty framing remains slightly inflated in some sections of the manuscript. The identification of TAB2 as an OTUB1-regulated substrate in microglia is biologically interesting and mechanistically relevant, but the overall conceptual advance remains an extension and refinement of existing ubiquitin-dependent TLR signaling paradigms rather than a fundamentally new signaling principle. Terms such as "pivotal regulator" should therefore be used with caution.

**Response:** We toned down the expression and removed "pivotal", line 28, 410.

Referee #2 (Remarks for Author):

The authors have sufficiently addressed my concerns. I suggested to accept this manuscript.

9th Jun 2026

Dear Prof. Wang,

We are pleased to inform you that your manuscript is accepted for publication and is now being sent to our publisher to be included in the next available issue of EMBO Molecular Medicine.

Your manuscript will be processed for publication by EMBO Press. It will be copy edited and you will receive page proofs prior to publication. Please note that you will be contacted by Springer Nature Author Services to complete licensing and payment information.

You may qualify for financial assistance for your publication charges - either via a Springer Nature fully open access agreement or an EMBO initiative. Check your eligibility: https://link.springer.com/journal/44321/how-to-publish-with-us

Should you be planning a Press Release on your article, please notify the production team via this form in order to coordinate publication and release dates: https://forms.cloud.microsoft/e/eH4A8JVP6g

If you have any questions, please do not hesitate to contact the Editorial Office. Thank you for your contribution to EMBO Molecular Medicine.

Kind regards,
Jingyi

Jingyi Hou
Senior Editor
EMBO Molecular Medicine

------------------------------------------------

>>> Please note that it is EMBO Molecular Medicine policy for the transcript of the editorial process (containing referee reports and your response letter) to be published as an online supplement to each paper. If you do NOT want this, you will need to inform the Editorial Office via email immediately. More information is available here: https://link.springer.com/partners/embo-press/editorial-policies#Peer%20review

## EMBO Press Author Checklist


| | |
|---|---|
| Corresponding Author Name: Xu Wang | |
| Journal Submitted to: EMBO Molecular Medicine | |
| Manuscript Number: EMM-2025-23062 | |


**USEFUL LINKS FOR COMPLETING THIS FORM**
The EMBO Journal - Author Guidelines
EMBO Reports - Author Guidelines
Molecular Systems Biology - Author Guidelines
EMBO Molecular Medicine - Author Guidelines

**Reporting Checklist for Life Science Articles (updated January**
This checklist is adapted from Materials Design Analysis Reporting (MDAR) Checklist for Authors. MDAR establishes a minimum set of requirements in transparent reporting in the life sciences (see Statement of Task: 10.31222/osf.io/9sm4x). Please follow the journal's guidelines in preparing your
**Please note that a copy of this checklist will be published alongside your article.**

**Abridged guidelines for figures**
**1. Data**
The data shown in figures should satisfy the following conditions:
- the data were obtained and processed according to the field's best practice and are presented to reflect the results of the experiments in an accurate and unbiased manner.
- ideally, figure panels should include only measurements that are directly comparable to each other and obtained with the same assay.
- plots include clearly labeled error bars for independent experiments and sample sizes. Unless justified, error bars should not be shown for technical
- if n<5, the individual data points from each experiment should be plotted. Any statistical test employed should be justified.
- Source Data should be included to report the data underlying figures according to the guidelines set out in the authorship guidelines on Data

**2. Captions**
Each figure caption should contain the following information, for each panel where they are relevant:
- a specification of the experimental system investigated (eg cell line, species name).
- the assay(s) and method(s) used to carry out the reported observations and measurements.
- an explicit mention of the biological and chemical entity(ies) that are being measured.
- an explicit mention of the biological and chemical entity(ies) that are altered/varied/perturbed in a controlled manner.
- the exact sample size (n) for each experimental group/condition, given as a number, not a range;
- a description of the sample collection allowing the reader to understand whether the samples represent technical or biological replicates (including how many animals, litters, cultures, etc.).
- a statement of how many times the experiment shown was independently replicated in the laboratory.
- definitions of statistical methods and measures:
  - common tests, such as t-test (please specify whether paired vs. unpaired), simple $\chi^2$ tests, Wilcoxon and Mann-Whitney tests, can be unambiguously identified by name only, but more complex techniques should be described in the methods section;
  - are tests one-sided or two-sided?
  - are there adjustments for multiple comparisons?
  - exact statistical test results, e.g., P values = x but not P values < x;
  - definition of 'center values' as median or average;
  - definition of error bars as s.d. or s.e.m.

**Please complete ALL of the questions below.**
**Select "Not Applicable" only when the requested information is not relevant for your study.**

### Materials

| Newly Created Materials | Information included in the manuscript? | In which section is the information available? (Reagents and Tools Table, Materials and Methods, Figures, Data Availability Section) |
|---|---|---|
| New materials and reagents need to be available; do any restrictions apply? | Yes | Reagents and Tools Table, Materials and Methods |

| Antibodies | Information included in the manuscript? | In which section is the information available? (Reagents and Tools Table, Materials and Methods, Figures, Data Availability Section) |
|---|---|---|
| For **antibodies** provide the following information:<br>- Commercial antibodies: RRID (if possible) or supplier name, catalogue number and or/clone number<br>- Non-commercial: RRID or citation | Yes | Reagents and Tools Table, Materials and Methods |

| DNA and RNA sequences | Information included in the manuscript? | In which section is the information available? (Reagents and Tools Table, Materials and Methods, Figures, Data Availability Section) |
|---|---|---|
| **Short novel DNA or RNA including primers, probes**: provide the sequences. | Yes | Reagents and Tools Table, Materials and Methods |

| Cell materials | Information included in the manuscript? | In which section is the information available? (Reagents and Tools Table, Materials and Methods, Figures, Data Availability Section) |
|---|---|---|
| **Cell lines:** Provide species information, strain. Provide accession number in repository **OR** supplier name, catalog number, clone number, and/**OR** RRID. | Yes | Reagents and Tools Table, Materials and Methods |
| **Primary cultures:** Provide species, strain, sex of origin, genetic modification status. | Yes | Materials and Methods |
| Report if the cell lines were recently **authenticated** (e.g., by STR profiling) and tested for mycoplasma contamination. | Not Applicable | |

| Experimental animals | Information included in the manuscript? | In which section is the information available? (Reagents and Tools Table, Materials and Methods, Figures, Data Availability Section) |
|---|---|---|
| **Laboratory animals or Model organisms:** Provide species, strain, sex, age, genetic modification status. Provide accession number in repository **OR** supplier name, catalog number, clone number, **OR** RRID. | Yes | Reagents and Tools Table, Materials and Methods |
| **Animal observed in or captured from the field:** Provide species, sex, and age where possible. | Not Applicable | |
| Please detail **housing and husbandry conditions**. | Yes | Materials and Methods |

| Plants and microbes | Information included in the manuscript? | In which section is the information available? (Reagents and Tools Table, Materials and Methods, Figures, Data Availability Section) |
|---|---|---|
| **Plants:** provide species and strain, ecotype and cultivar where relevant, unique accession number if available, and source (including location for collected wild specimens). | Not Applicable | |
| **Microbes:** provide species and strain, unique accession number if available, and source. | Not Applicable | |

| Human research participants | Information included in the manuscript? | In which section is the information available? (Reagents and Tools Table, Materials and Methods, Figures, Data Availability Section) |
|---|---|---|
| If collected and within the bounds of privacy constraints report on age, sex and gender or ethnicity for all study participants. | Not Applicable | |

| Core facilities | Information included in the manuscript? | In which section is the information available? (Reagents and Tools Table, Materials and Methods, Figures, Data Availability Section) |
|---|---|---|
| If your work benefited from core facilities, was their service mentioned in the acknowledgments section? | Yes | Acknowledgment |

### Design

| Study protocol | Information included in the manuscript? | In which section is the information available? (Reagents and Tools Table, Materials and Methods, Figures, Data Availability Section) |
|---|---|---|
| If study protocol has been **pre-registered, provide DOI in the manuscript**. For clinical trials, provide the trial registration number **OR** cite DOI. | Not Applicable | |
| Report the **clinical trial registration number** (at ClinicalTrials.gov or equivalent), where applicable. | Not Applicable | |

| Laboratory protocol | Information included in the manuscript? | In which section is the information available? (Reagents and Tools Table, Materials and Methods, Figures, Data Availability Section) |
|---|---|---|
| Provide DOI OR other citation details if **external detailed step-by-step protocols** are available. | Yes | Materials and Methods |

| Experimental study design and statistics | Information included in the manuscript? | In which section is the information available? (Reagents and Tools Table, Materials and Methods, Figures, Data Availability Section) |
|---|---|---|
| Include a statement about **sample size** estimate even if no statistical methods were used. | Yes | Figures and figure legends |
| Were any steps taken to minimize the effects of subjective bias when allocating animals/samples to treatment (e.g. **randomization procedure**)? If yes, have they been described? | Yes | Materials and Methods |
| Include a statement about **blinding** even if no blinding was done. | Yes | Materials and Methods |
| Describe **inclusion/exclusion criteria** if samples or animals were excluded from the analysis. Were the criteria pre-established?<br>If sample or data points were omitted from analysis, report if this was due to attrition or intentional exclusion and provide justification. | Yes | Materials and Methods |
| For every figure, are **statistical tests** justified as appropriate? Do the data meet the assumptions of the tests (e.g., normal distribution)? Describe any methods used to assess it. Is there an estimate of variation within each group of data? Is the variance similar between the groups that are being statistically compared? | Yes | Materials and Methods,Statistics table |

| Sample definition and in-laboratory replication | Information included in the manuscript? | In which section is the information available? (Reagents and Tools Table, Materials and Methods, Figures, Data Availability Section) |
|---|---|---|
| In the figure legends: state number of times the experiment was **replicated** in laboratory. | Yes | Figure legends, Materials and Methods |
| In the figure legends: define whether data describe **technical or biological replicates**. | Yes | Figure legends |

## Ethics

| Ethics | Information included in the manuscript? | In which section is the information available? (Reagents and Tools Table, Materials and Methods, Figures, Data Availability Section) |
|---|---|---|
| Studies involving **human participants**: State details of **authority granting ethics approval** (IRB or equivalent committee(s), provide reference number for approval. | Not Applicable | |
| Studies involving **human participants**: Include a statement confirming that **informed consent** was obtained from all subjects and that the experiments conformed to the principles set out in the WMA Declaration of Helsinki and the Department of Health and Human Services Belmont Report. | Not Applicable | |
| Studies involving **human participants: For** publication of **patient photos**, include a statement confirming that consent to publish was obtained. | Not Applicable | |
| Studies involving experimental **animals**: State details of **authority granting ethics approval** (IRB or equivalent committee(s), provide reference number for approval. Include a statement of compliance with ethical regulations. | Yes | Materials and Methods |
| Studies involving **specimen and field samples:** State if relevant **permits** obtained, provide details of authority approving study; if none were required, explain why. | Not Applicable | |

| Dual Use Research of Concern (DURC) | Information included in the manuscript? | In which section is the information available? (Reagents and Tools Table, Materials and Methods, Figures, Data Availability Section) |
|---|---|---|
| Could your study fall under dual use research restrictions? Please check biosecurity documents and list of **select agents and toxins** (CDC): https://www.selectagents.gov/sat/list.htm | Not Applicable | |
| If you used a select agent, is the security level of the lab appropriate and reported in the manuscript? | Not Applicable | |
| If a study is subject to dual use research of concern regulations, is the name of the **authority granting approval and reference number** for the regulatory approval provided in the manuscript? | Not Applicable | |

## Reporting

The MDAR framework recommends adoption of discipline-specific guidelines, established and endorsed through community initiatives. Journals have their own policy about requiring specific guidelines and recommendations to complement MDAR.

| Adherence to community standards | Information included in the manuscript? | In which section is the information available? (Reagents and Tools Table, Materials and Methods, Figures, Data Availability Section) |
|---|---|---|
| State if relevant guidelines or checklists (e.g., **ICMJE, MIBBI, ARRIVE, PRISMA**) have been followed or provided. | Not Applicable | |
| For **tumor marker prognostic studies,** we recommend that you follow the **REMARK** reporting guidelines (see link list at top right). See author guidelines, under 'Reporting Guidelines'. Please confirm you have followed these guidelines. | Not Applicable | |
| For **phase II and III randomized controlled trials**, please refer to the **CONSORT** flow diagram (see link list at top right) and submit the CONSORT checklist (see link list at top right) with your submission. See author guidelines, under 'Reporting Guidelines'. Please confirm you have submitted this list. | Not Applicable | |

## Data Availability

| Data availability | Information included in the manuscript? | In which section is the information available? (Reagents and Tools Table, Materials and Methods, Figures, Data Availability Section) |
|---|---|---|
| Have **primary datasets** been deposited according to the journal's guidelines (see 'Data Deposition' section) and the respective accession numbers provided in the Data Availability Section? | Not Applicable | |
| Were **human clinical and genomic datasets** deposited in a public access-controlled repository in accordance to ethical obligations to the patients and to the applicable consent agreement? | Not Applicable | |
| Are **computational models** that are central and integral to a study available without restrictions in a machine-readable form? Were the relevant accession numbers or links provided? | Not Applicable | |
| If publicly available data were reused, provide the respective **data citations in the reference list**. | Not Applicable | |
