# Supplementary material for: OTUB1 non-canonically inhibits TAB2 ubiquitination to govern microglia-mediated neuroinflammation
Source: EMBO Mol Med. 2026 Jul 2;18(8):3137–56. doi: 10.1038/s44321-026-00473-x (PMC13470327; doi:10.1038/s44321-026-00473-x)
Supplement: Supplementary file 9 [file 44321_2026_473_MOESM6_ESM.zip › EMM-2025-23062_SourceDataForFigure 4/4E/4E.pdf]

Colocalization_of_C1-Series003_overlay.tif_versus_C2-Series003_overlay.tif_1342926306.pdf

Li - Ch1

256.0 x 256.0

Channel 2
C2-Series003_overlay.tif

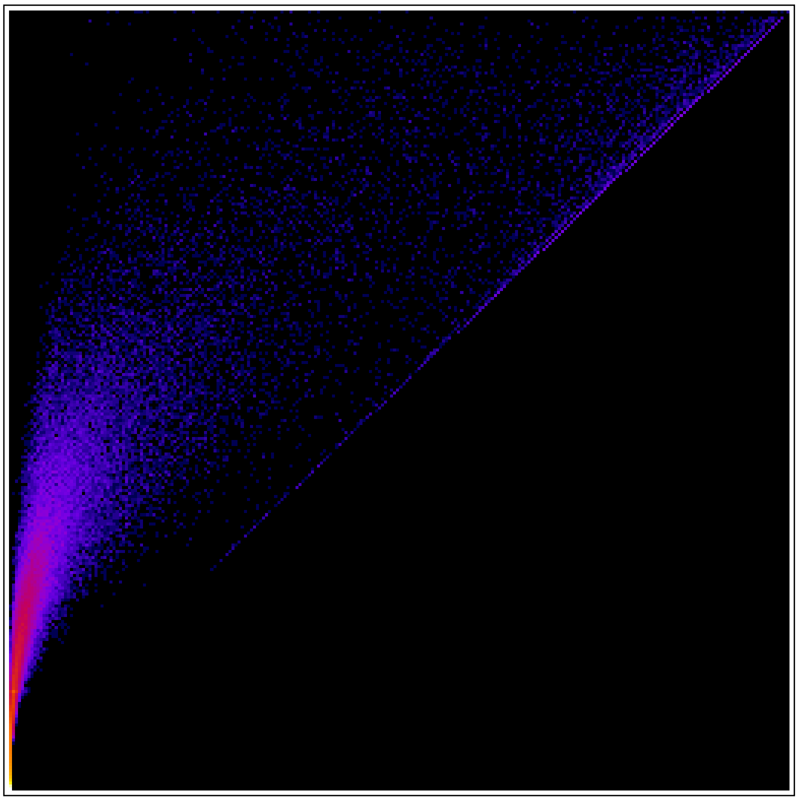

Channel 1
C1-Series003_overlay.tif

Li - Ch2

256.0 x 256.0

Channel 2
C2-Series003_overlay.tif

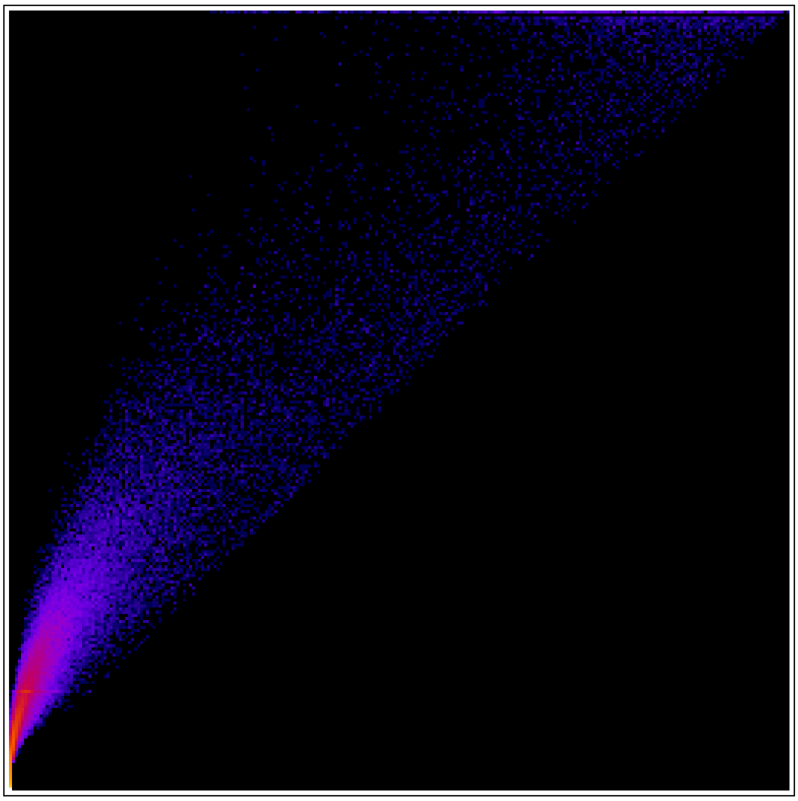

Channel 1
C1-Series003_overlay.tif

2D intensity histogram

256.0 x 256.0

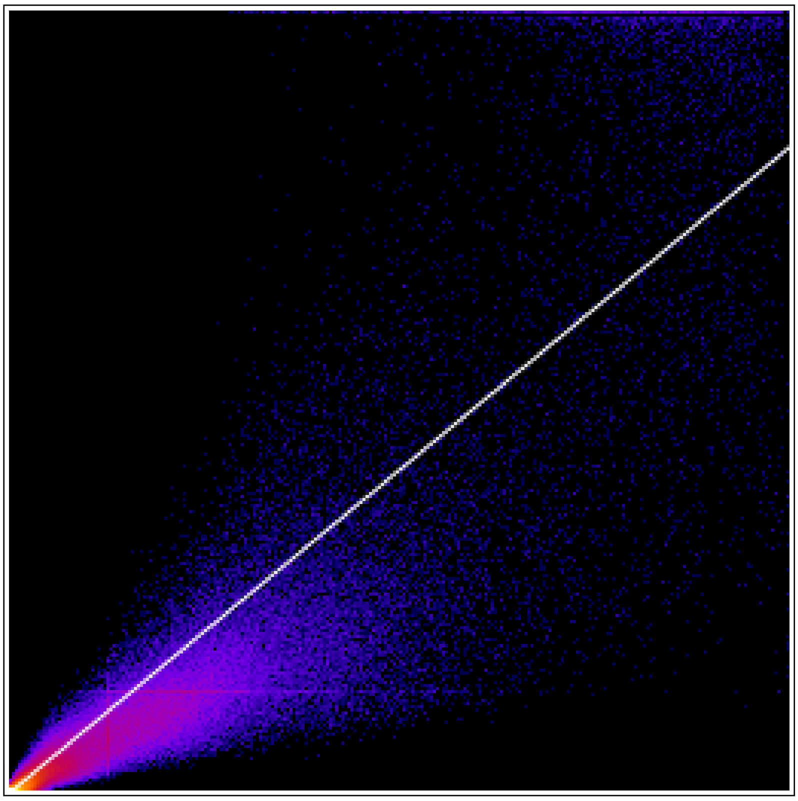

Channel 2
C2-Series003_overlay.tif

Channel 1
C1-Series003_overlay.tif

Smoothed & shuffled channel 1

1024.0 x 1024.0

Channel 2
C2-Series003_overlay.tif

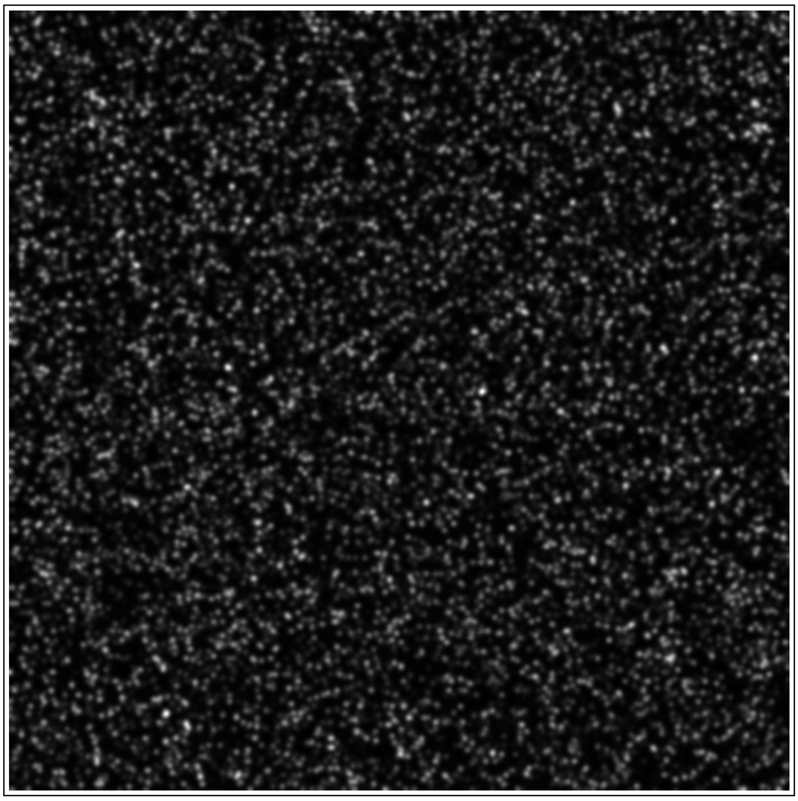

Channel 1
C1-Series003_overlay.tif

Channel 1 (Max Projection)

1024.0 x 1024.0

Channel 2
C2-Series003_overlay.tif

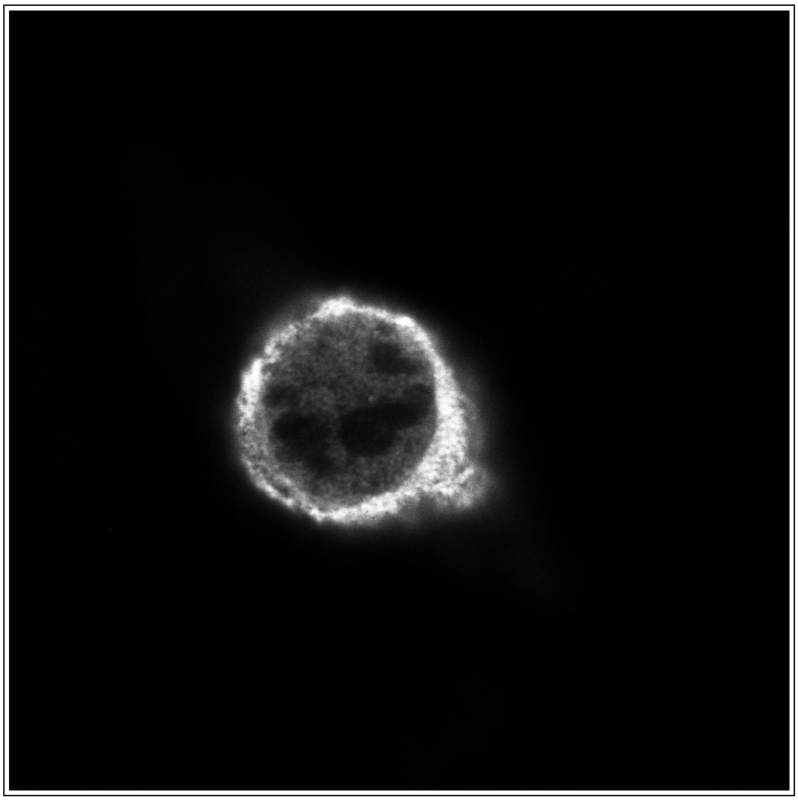

Channel 1
C1-Series003_overlay.tif

Channel 2 (Max Projection)
1024.0 x 1024.0

Channel 2
C2-Series003_overlay.tif

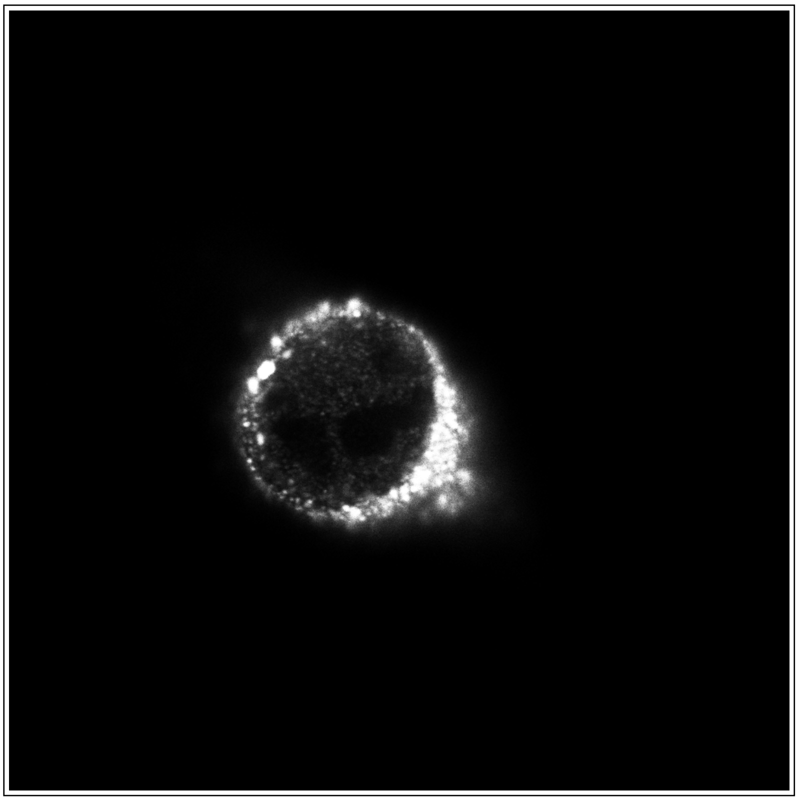

Channel 1
C1-Series003_overlay.tif

Warning! Zero-zero ratio too high - The ratio between zero-zero pixels and other pixels is large: 0.57. Maybe you should use a ROI.
Warning! y-intercept far from zero - The ratio of the y-intercept of the auto threshold regression line to the mean value of Channel 2 is high. This means the y-intercept is far from zero, implying a significant positive or negative zero offset in the image data intensities. Maybe you should use a ROI. Maybe do a background subtraction in both channels. Make sure you didn't clip off the low intensities to zero. This might not affect Pearson's correlation values very much, but might harm other results.
Warning! Threshold of ch. 1 too high - Too few pixels are taken into account for above-threshold calculations. The threshold is above the channel's mean.
Warning! Threshold of ch. 2 too high - Too few pixels are taken into account for above-threshold calculations. The threshold is above the channel's mean.
Coloc_Job_Name: Colocalization_of_C1-Series003_overlay.tif_versus_C2-Series003_overlay.tif_1342926306
% zero-zero pixels: 56.73
% saturated ch1 pixels: 0.02
% saturated ch2 pixels: 0.26
Channel 1 Max: 255.000
Channel 2 Max: 255.000
Channel 1 Min: 0.000
Channel 2 Min: 0.000
Channel 1 Mean: 8.793

Channel 2 Mean: 5.901
Channel 1 Integrated (Sum) Intensity: 9219699.000
Channel 2 Integrated (Sum) Intensity: 6187140.000
Mask Type Used: none
Mask ID Used: 1342926306
m (slope): 0.83
b (y-intercept): -1.42
b to y-mean ratio: -0.24
Ch1 Max Threshold: 167.00
Ch2 Max Threshold: 138.00
Threshold regression: Costes
Pearson's R value (no threshold): 0.89
Pearson's R value (below threshold): 0.85
Pearson's R value (above threshold): -0.03
Li's ICQ value: 0.487
Spearman's rank correlation value: 0.68804343
Spearman's correlation t-statistic: 970.9045
t-statistic degrees of freedom: 1048574.000
Manders' M1 (Above zero intensity of Ch2): 0.953
Manders' M2 (Above zero intensity of Ch1): 1.000
Manders' tM1 (Above autothreshold of Ch2): 0.263
Manders' tM2 (Above autothreshold of Ch1): 0.385
Kendall's Tau-b rank correlation value: 0.6479
Costes P-Value: 1.00
Costes Shuffled Mean: -0.00
Costes Shuffled Std.D.: 0.01
Ratio of rand. Pearsons >= actual Pearsons value : 0.00
